# Size Sound Symbolism Modulates Linguistic Processing: An ERP Study

**DOI:** 10.1111/psyp.70190

**Published:** 2025-11-28

**Authors:** Sarah Glim, Ralf Rummer

**Affiliations:** ^1^ Department of Psychology University of Kassel Kassel Germany

**Keywords:** EEG, LPC, N400, size perception, sound symbolism

## Abstract

The size of physical objects is systematically associated with specific speech sounds used to refer to these objects. This phenomenon, termed size sound symbolism, has been demonstrated with a number of different behavioral measures such as word rating or word selection tasks. Yet, there is little data on where and how such sound‐symbolic associations come into play within the brain's cognitive processing hierarchy. In the present EEG study, we investigated whether the neural activation of associations based on size sound symbolism can be automatic by nature. Participants were presented with small or large novel visual objects (greebles), followed by small‐sounding or large‐sounding (containing the letter <i> or <a>) fictional greeble names. We found, in accordance with our hypotheses, that the processing of sound‐symbolically congruent names, compared to incongruent names, elicited a reduced N400 ERP component, in particular with regard to the small greebles. An additional exploratory analysis revealed an effect of size sound symbolism also in a subsequent time window, capturing a late positive component. These findings were evident in the absence of any task demands or conscious awareness related to sound symbolism. We argue that the greebles' presentation entailed an automatic activation of sound‐symbolically associated linguistic information, which in turn facilitated the subsequent linguistic processing of sound‐symbolically matching input, followed by stronger engagement of memory functions. The present study thus demonstrates that size sound symbolism is an inherent component of the brain's information processing system rather than a product of deliberate decision or response mechanisms and that it thereby exerts a significant influence on how we experience the world around us.

## Impact Statement

Our findings demonstrate the neural relevance of sound‐symbolic associations between novel visual objects of different sizes and different fictional object names. We show that sound‐symbolically congruent versus incongruent information elicits different processing in the human brain—without any task demands related to sound symbolism and without participants' conscious awareness of it—and that size sound symbolism is thus deeply enrooted in the brain's automatic information processing system.

Mainstream linguistics has long assumed that the relationship between the sound of words and their meaning is essentially arbitrary (see de Saussure 1916). The phenomenon of *sound symbolism* or *phonological iconicity*, however, highlights the existence of nonarbitrary associations between certain language sounds and the semantic concepts that they refer to (with this idea dating back at least to Plato's *Cratylus* [Sedley 2003]; for reviews, see Imai and Kita 2014; Lockwood and Dingemanse 2015; Nuckolls 1999; Perniss et al. 2010; Sidhu 2025; Sidhu and Pexman 2018). Such form–meaning correspondences have been reported with regard to artificial nonwords and a multitude of existing languages (for a respective analysis of almost two‐thirds of the world's languages, see Blasi et al. 2016), and they are by now believed to be an important linguistic design principle, facilitating language acquisition and driving language evolution (e.g., Imai and Kita 2014; Imai et al. 2008; Kantartzis et al. 2011; Lockwood et al. 2016; Nygaard et al. 2009).

One of the best‐known examples of sound symbolism was provided by Köhler (1929), who suggested that participants consistently associate a rounded visual shape with the nonword *baluma* and a spiky visual shape with the nonword *takete*—a finding that was later popularized under the name *bouba*/*kiki* effect (see Ramachandran and Hubbard 2001) and that strikingly illustrates the existence of associations between signifier and signified in the domain of visual shape. Apart from shape, research to date has identified and dissected several further domains of sound symbolism, among them, for instance, valence (e.g., Rummer and Schweppe 2019; Rummer et al. 2014; Yu et al. 2021), color (e.g., Johansson et al. 2020; Kim et al. 2018; Mok et al. 2019), motion (e.g., Cuskley 2013; Shinohara et al. 2020; Zhao and Wu 2025), and size (see below).

In the same year that Köhler reported his observation on shape sound symbolism, Sapir (1929) demonstrated that participants tend to associate the nonword *mil* with a small object and the nonword *mal* with a large object, thereby coining the well‐known *mil*/*mal* effect. Since then, the existence and cognitive relevance of *magnitude* or *size sound symbolism* has been confirmed with regard to a number of phonetic factors by several other authors (but see Sidhu and Pexman 2023, for a report of null results), who examined, for example, the size‐related interpretation of nonwords by speakers of various languages (English, Chinese, Korean, and Japanese: Shinohara and Kawahara 2010; Hungarian and German: Elsen et al. 2021), statistical regularities among size adjectives within the English lexicon (Winter and Perlman 2021; for regularities on a global scale, i.e., across linguistic lineages and continents, see Blasi et al. 2016), and the impact of size‐sound‐symbolic pairings on recall from associative memory (Preziosi and Coane 2017).

In an insightful series of experiments on size sound symbolism, Thompson and Estes (2011) presented participants with novel visual objects, so‐called greebles, of different sizes and asked them to select the most fitting name for each greeble from a list of different nonwords, which varied in the number of phonemes that had been associated with smallness versus largeness in previous research. The results showed that size sound symbolism operates in a graded fashion, with participants preferring increasingly larger‐sounding names for increasingly larger greebles. The authors interpreted this finding in terms of a crossmodal explanation of sound symbolism (see also Ramachandran and Hubbard 2001), according to which the names of objects are directly related to physical properties of these objects. It was argued that such crossmodal matching might have emerged from the representation of gestures, with the mouth and vocal tract producing vocal gestures that mimic an object's physical properties, and/or from sound pitch, with smaller objects resonating at and thus being related to higher (acoustic) frequencies (see Ohala 1995). The latter hypothesis in particular has often been used to explain the size‐sound‐symbolic *mil*/*mal* effect, based on the argument that high front vowels—as in *mil*—produce resonant frequencies that are characteristic of small vocal tracts (and that might therefore be uttered by nonhuman species to simulate small size and thereby submissiveness). For a contemporary evaluation of the different explanations of size sound symbolism, see Ekström (2022).

It should be noted at this point, though, that most of the available experimental data on (size) sound symbolism have been obtained with behavioral measures such as explicit name rating or name selection tasks (e.g., Elsen et al. 2021; Shinohara and Kawahara 2010; Thompson and Estes 2011; for an implicit behavioral task, see Parise and Spence 2012). These measures clearly demonstrate the existence of sound symbolism but typically fail to provide direct evidence of the proposed crossmodal associations in the physical processing system, leaving open the question of where and how such associations come into play within the brain's neuro‐cognitive hierarchy. Alternatives range from an inherent and automatic coactivation of sound‐symbolic associations during initial stimulus processing on the one hand to the emergence of such associations only as a product of higher‐order conscious and deliberate decision processes on the other hand. Approaching this issue from a behavioral perspective using an implicit association test (IAT), Parise and Spence (2012) demonstrated that participants respond faster to sound‐symbolically congruent, compared to incongruent, assignments between response keys and different stimuli of interest (e.g., a small circle and the nonword *mil*; a large circle and the nonword *mal*). As this pattern was observed even within the fastest response time bins, the authors argued against an explanation based only on explicit cognitive strategies and for the influence of automatic processes. Importantly, though, the proposed automatic processing mechanisms might be accessed more directly with neuroscientific methods, especially the method of electroencephalography (EEG), which allows for the evaluation of stimulus‐related brain activity at a high temporal resolution.

So far, only a few studies have investigated sound‐symbolic processing mechanisms using EEG. Kovic et al. (2010) asked participants to learn associations between visually presented novel objects and auditorily presented nonword names, which were either sound‐symbolically congruent or incongruent with regard to the dimension of shape. That is, a round‐sounding or sharp‐sounding name was assigned to a rounded or pointed visual object category. An analysis of event‐related potentials (ERPs) revealed that the categorization of visual objects that had been associated with sound‐symbolically congruent, compared to incongruent, names went along with a stronger negativity between 140 and 180 ms after stimulus onset at occipital electrode sites. This finding was interpreted as reflecting visual–auditory feature integration during sensory processing, with a better consolidated integration of the previously learnt multisensory associations in the congruent condition. In another EEG study (Asano et al. 2015), participants, in this case preverbal infants, were presented with a rounded or spiky visual shape, followed by a sound‐symbolically congruent or incongruent auditory nonword. Here, an analysis of ERPs showed that the congruent, compared to incongruent, nonwords elicited a reduced N400 amplitude at central electrode sites. Investigating sound‐symbolic dimensions beyond shape, Glim and Hillje (2025) presented participants with schematic drawings of cars—a cabriolet (categorized, among other aspects, as small, thin, and light) and a sport utility vehicle (SUV; having the opposite characteristics)—followed by a sound‐symbolically congruent or incongruent visually presented fictional car name. Here again, the congruent and incongruent pairings differed with regard to the N400 component, with a reduced N400 amplitude at parietal electrode sites during the processing of the congruent names, especially in relation to the cabriolet.

It thus appears that the N400 component is a particularly promising marker of sound‐symbolic processing in the human brain. This component was first described more than 40 years ago (Kutas and Hillyard 1980). Kutas and Hillyard showed in their study that the presentation of semantically inappropriate and thus unexpected words within sentence structures elicited a negative brain potential with a peak at about 400 ms after word onset. The detected component was proposed to indicate the reprocessing of semantically anomalous information or, in other words, the second look that participants give to the deviant input to try to make sense of it (Kutas and Hillyard 1980). Since then, the N400 component has been examined in a multitude of other EEG studies that have gradually expanded our understanding of the contexts in which it occurs and, consequently, of the neural mechanisms that it most likely represents (for a review, see Kutas and Federmeier 2011). All things considered, the N400 seems to reflect the processing of meaning with regard to a wide range of linguistic and nonlinguistic stimuli or, more precisely, “the activity in a multimodal long‐term memory system that is induced by a given input stimulus during a delimited time window as meaning is dynamically constructed” (Kutas and Federmeier 2011, p. 640). Interestingly, Kutas and Federmeier furthermore argued that such stimulus‐induced activity is reduced when the relevant information is already preactivated in semantic memory and that a reduction of the N400's amplitude is thus indicative of a state in which the respective information has already been active at the time of stimulus processing. This facilitating state seems to be created, in particular, by a supportive/constraining context (e.g., a preceding semantically associated word) that allows for the generation of specific predictions about the upcoming stimulus input (Lau et al. 2013; Szewczyk and Schriefers 2018), with such predictive processing presumably being a core feature of the brain's general mode of operation (e.g., Bubic et al. 2010; Clark 2013). In fact, a number of studies have shown that the N400 component is modulated by the associative relationship between stimuli, with a high associative strength (e.g., based on past experiences of stimulus contiguity) resulting in a reduced N400 amplitude (Ortu et al. 2013; Rhodes and Donaldson 2008).

Following this interpretation, one might then argue that the reduced N400 amplitude that has been observed during the processing of sound‐symbolically congruent stimuli (Asano et al. 2015; Glim and Hillje 2025) was a consequence of the high associative strength between these stimuli. The sound‐symbolic association presumably enabled the brain to use the context of the initially presented visual stimulus (e.g., the car in Glim and Hillje 2025) for the generation of predictions about and consequently facilitated processing of the matching linguistic information (e.g., the car's name). The discussed EEG studies thus indicate that sound symbolism can influence linguistic stimulus processing—and notably so in an automatic manner, that is, without participants explicitly focusing on the formation and/or evaluation of sound‐symbolic matches. Still, the extent to which this conclusion is specific to the investigated experimental factors or generalizes beyond them remains an open question.

In the present EEG study, we expanded the available knowledge on neural processing mechanisms to size sound symbolism, which potentially contributed, to a hitherto unknown extent, to the car type manipulation reported in Glim and Hillje (2025) but has not been examined in isolation. In each trial of the present study, participants observed a small or large greeble (see Thompson and Estes 2011), followed by a small‐sounding or large‐sounding fictional greeble name, and attended to size‐unrelated features of these stimuli. In accordance with the classical *mil*/*mal* effect of size sound symbolism (Sapir 1929), names were created by contrasting the vowel letter <i> with the vowel letter <a>. This manipulation further expanded the data collected in the study of Glim and Hillje (2025), where <i> was contrasted with <o>. Generalizing the results of this previous study, we expected an interaction effect between greeble size and greeble name, with a reduced/less negative N400 amplitude during greeble name processing for sound‐symbolically congruent, compared to incongruent, greeble–name pairings. Considering that the respective N400 difference in Glim and Hillje (2025) was more pronounced for the cabriolet than for the SUV—with these stimuli differing along several dimensions beyond size—we were, additionally, interested in seeing whether this pattern transferred to the present stimuli, with a potentially more pronounced difference for small greebles than for large greebles. This hypothesis was furthermore motivated by previous work on sound symbolism in existing languages that revealed a global association between smallness and the vowel <i> but no comparable effect for the concept of largeness (Blasi et al. 2016). Beyond testing the outlined hypotheses, we also carried out exploratory analyses to examine whether size sound symbolism influences ERP components other than the N400. Evidence for a modulation of neural processing by automatically activated (unconscious and task‐irrelevant) sound‐symbolic associations would tie the phenomenon of size sound symbolism to the human brain's information processing system, suggesting that such symbolism could play a meaningful role in how we naturally experience language within the context of the physical world around us.

## 1 Methods

### 1.1 Participants

Thirty‐one participants were recruited from the local student community. All participants reported to be German native speakers, right‐handed, and neurologically healthy, with normal or corrected‐to‐normal vision. Participants provided written informed consent and were compensated with 10€/h or partial course credit. They were all naïve with regard to the study's hypotheses (confirmed by a postexperimental survey item; see Procedure), with many participants expressing surprise upon learning about the study's focus of interest during debriefing. One participant was excluded due to low (< 70% correct) performance on the attention check questions (see Design and Materials), which resulted in a final sample of 30 participants (24 women, 6 men; age: *M* = 22 years, SD = 5 years, range = 18–38 years; performance on the attention check questions: *M* = 87.19% correct, SD = 8.66% correct, range = 71.88%–100% correct). The final sample size was based on our standard laboratory protocol for ERP studies and corresponded to the size assessed in Glim and Hillje (2025; see also Glim et al. 2023, 2024, 2025). The study followed local institutional guidelines and was conducted in accordance with the Declaration of Helsinki.

### 1.2 Design and Materials

The experiment employed a 2 × 2 within‐subjects design with the factors *greeble size* (small vs. large) and *vowels* used in the greeble names (<i> vs. <a>). The resulting four conditions were realized in 80 greeble–name combination trials each, with the experiment thus consisting of 320 trials in total, plus eight initial training trials.

**FIGURE 1:**
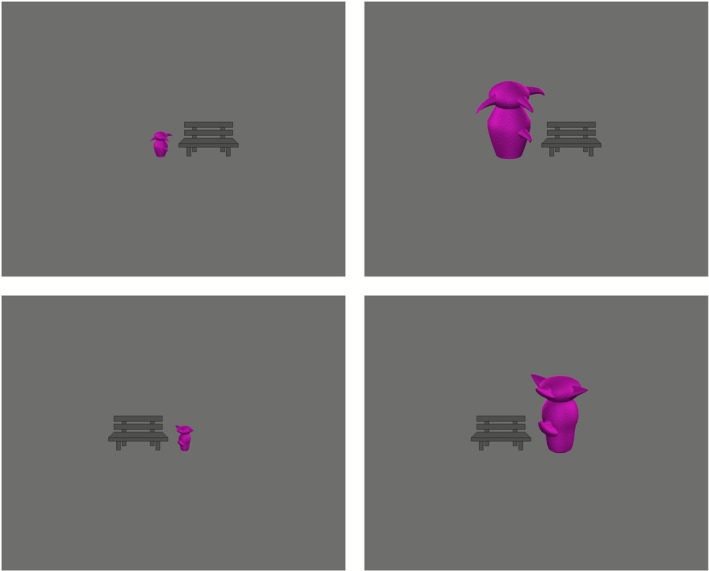
Stimulus examples. In each trial, participants saw either a small greeble or a large greeble (followed by the greeble's name). Half of the greebles were presented to the left of a schematic drawing of a bench; the other half were presented to the right of the bench.

The greeble images were downloaded from the Tarrlab website, courtesy of Michael J. Tarr, Carnegie Mellon University, http://www.tarrlab.org/ (see e.g., Gauthier and Tarr 1997; Gauthier et al. 1998). We selected the 80 images from the Greebles‐2‐0‐symmetric dataset that were displayed with regard to viewpoint ‘v1’. The original greebles were used in the large greeble size condition; the small greeble size condition was then created by shrinking the greebles to 33% of their original size. Each purple greeble was presented next to a schematic drawing of a dark gray bench, which served as a reference for size and distance to the observer (see also Thompson and Estes 2011, who presented the greebles next to images of a cow and an abstract human figure). Half of the greebles (40 individual greebles, once in small and once in large) appeared to the left of the bench, while the other half appeared to the right of it. The different greebles of one side, left or right, were always presented at the same position and were always facing the screen's vertical midline, which was in between greeble and bench (see Figure 1 for stimulus examples). In addition, we selected eight greeble images from the Greebles‐2‐0‐asymmetric dataset for usage during training (viewpoint ‘v1’; half of them presented in their original size and the other half shrunk to 33%; half of them presented to the left of the bench and the other half presented to the right of it).

For the greeble names, we created 80 pairs of German pseudowords, following the pattern CVCVC. The three consonant spaces were filled so that each of 20 consonant letters (<b/c/d/f/g/h/j/k/l/m/n/p/q/r/s/t/v/w/x/z>) appeared equally often at each position and did not repeat within a given pseudoword. The two vowel spaces were filled with either twice the letter <i> or twice the letter <a>, which resulted, for example, in *Zitil* and *Zatal* as one pseudoword pair.^1^ Each pseudoword pair was assigned to one of the 80 individual greebles (presented once in small and once in large, see above). In addition, we created eight pseudowords for usage during training, with half of them containing the letter <i> and the other half containing the letter <a>. A list of all created pseudowords as well as their assignment to the different greeble configurations can be found in the present article's online Supporting Information.

Thirty‐two greeble–name combinations, plus the eight combinations used during training, were followed by a question about the presented stimuli. The questions were intended to secure a constant level of attention without disclosing the experiment's actual manipulations of interest or imposing any task demands related to size sound symbolism. 50% of the questions were related to the just‐presented greeble (either *From your perspective, was the greeble standing to the left of the bench?* or *From your perspective, was the greeble standing to the right of the bench?*), while the other 50% of questions were related to the just‐presented pseudoword (either *Did the majority of consonants within the greeble's name come from the first half of the alphabet [up to “M”]?* or *Did the majority of consonants within the greeble's name come from the second half of the alphabet [starting from “N”]?*). 50% of questions required a ‘yes’ response, while the other 50% of questions required a ‘no’ response. In the main task, the four different question types (relating to the greeble or to the pseudoword; requiring a ‘yes’ or a ‘no’ response) were used equally often with respect to trials of the four different conditions (small or large greeble; vowel letter <i> or <a> in the greeble's name; see the online Supporting Information). The questions appeared at irregular intervals. The participants' responses during training, but not during the main task, were followed by visual feedback. Participants were instructed to observe the presented greeble–name combinations carefully and to answer the questions as accurately as possible without being concerned with response speed.

### 1.3 Procedure

Each trial started with the presentation of a gray background screen (2500–3000 ms). Subsequently, a central black fixation cross was added (500 ms) and followed by another gray background screen (200 ms). We then presented the greeble and bench (1500 ms), another background screen (200 ms), and lastly, at the screen's center in black font, the greeble's name (800 ms). In trials that contained an attention check question, an additional background screen (200 ms) and then the question, displayed until a response was registered, ensued.

The different trials, and thus conditions, were presented in randomized order. A self‐paced pause was inserted after every 40 trials of the main task. Following the completion of all trials, participants provided demographic information, specified their assumptions about the study's purpose, and were debriefed. The study was programmed in Inquisit Lab (Millisecond Software LLC, USA, https://www.millisecond.com/).

### 1.4 Experimental Setup

The experiment was run in a darkened, sound‐attenuating testing booth (Desone Modulare Akustik, Germany, https://www.desone.de/). Participants were seated in front of a computer monitor, with a distance of 60 cm between the monitor's center and their eyes. The presentation screen's display resolution was 1280 × 1024 pixels. Behavioral responses, including those related to the provision of demographic information, were entered via a computer keyboard and mouse. Regarding the attention check questions, participants pressed the ‘A’ key with their left index finger to indicate a ‘yes’ response and the ‘L’ key with their right index finger to indicate a ‘no’ response.

The EEG setup consisted of an actiCHamp Plus amplifier and 64 active scalp electrodes, which were attached to an elastic electrode cap according to the 10% system (actiCAP slim/snap; Brain Products GmbH, Germany, https://www.brainproducts.com/). The electrode at FCz was used as the online reference, while an additional electrode at Fpz served as the ground electrode. Impedances were reduced with respect to a threshold of 20 kΩ. The sampling rate was 1000 Hz. We used the BrainVision Recorder software for EEG recording and the BrainVision Analyzer software for EEG preprocessing (Brain Products GmbH, Germany, https://www.brainproducts.com/).

### 1.5 Data Preprocessing and Analysis

EEG preprocessing included the following steps: (1) Filtering the data with a low cutoff at 0.1 Hz and a high cutoff at 35 Hz. (2) Constructing segments with a length of 1200 time points, ranging from 200 ms before to 999 ms after the onset of the greeble's name. (3) Manually rejecting all segments with prominent unsystematic noise in them, especially noise based on extensive body movements. (4) Running an independent component analysis (ICA) on all EEG channels and subtracting those components that were related to artifacts (e.g., eye movements, blinks, and muscle activity). (5) Rereferencing the data to the average of the signals from TP9 and TP10. (6) Performing a baseline correction with regard to the 200‐ms prestimulus interval. (7) Rejecting all remaining segments with artifacts based on visual inspection of the data combined with an objective detection procedure (maximal allowed voltage step: 30 μV/ms; maximal allowed absolute difference of values in intervals of 200 ms: 150 μV; minimal allowed amplitude: −150 μV; maximal allowed amplitude: 150 μV; lowest allowed activity [max − min] in intervals of 100 ms: 0.5 μV). Out of 80 trials per condition and participant, *M* = 75.63 trials (94.54%), SD = 4.07 trials, range = 60–80 trials survived data preprocessing and were entered into the following analyses, conducted in MATLAB (The MathWorks Inc., USA, https://www.mathworks.com/).

We averaged the preprocessed data over trials to obtain a data matrix of 64 EEG channels *×* 1200 time points per condition and participant. The N400 component was then analyzed as follows: Regarding the data's temporal dimension, we determined the trough of the ERP signal (averaged over channels, conditions, and participants) within a time window of 300–500 ms after the onset of the greeble's name, and we averaged the signals with regard to an interval of ±25 ms around this trough (for identical or slightly smaller activity‐centered N400 window sizes, see e.g., Bühler et al. 2017, who analyzed a time window of 454–494 ms, as well as Cummings et al. 2008; Hamm et al. 2002). Regarding the data's spatial dimension, we selected nine central and parietal EEG channels (C1, Cz, C2, CP1, CPz, CP2, P1, Pz, P2) for initial analysis, in accordance with the N400's typical centro‐parietal focus (Kutas and Federmeier 2011; Kutas and Hillyard 1989; for N400 studies with an identical channel selection, see e.g., Dudschig et al. 2016; Glim and Hillje 2025; Hoshino and Thierry 2012; Kröger et al. 2013; Zhang et al. 2019). The selected data were subjected to a repeated‐measures analysis of variance (ANOVA) with the experimental factors *greeble size* (small vs. large) and *vowels* used in the greeble names (<i> vs. <a>) as well as the spatial factors *posteriority* (central vs. centro‐parietal vs. parietal) and *laterality* (left vs. midline vs. right). The latter factors were included to allow for a more detailed assessment of the N400's spatial distribution, motivated by spatial discrepancies in previous sound symbolism studies (Asano et al. 2015; Glim and Hillje 2025). Spatial effects are only reported if they are significant in relation to interaction effects between the two experimental factors. Effects of interest were subsequently followed up by spatially more specific ANOVAs and one‐tailed paired‐sample *t*‐tests. In addition, exploratory analyses were performed as described below.

## 2 Results

**FIGURE 2:**
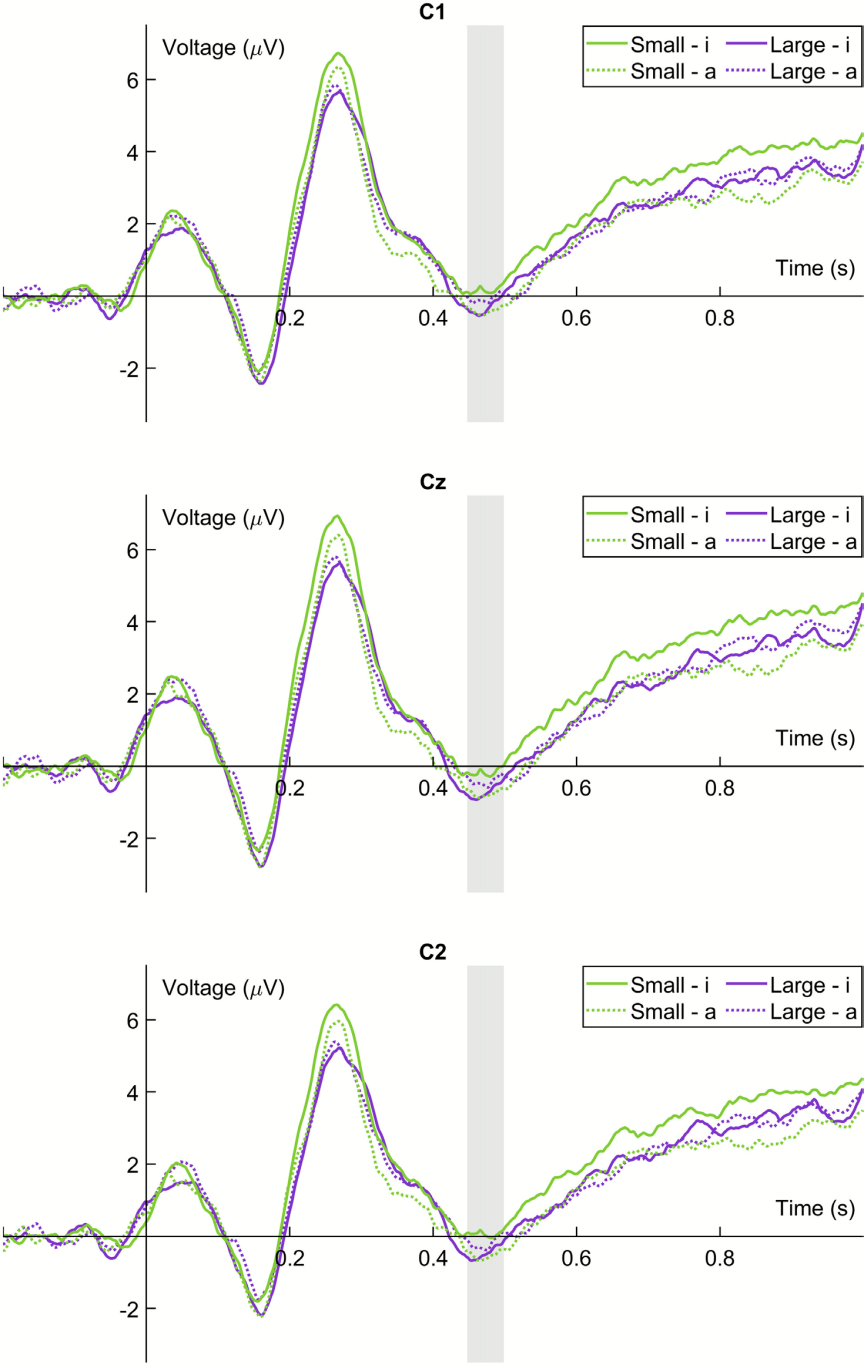
Event‐related potentials (ERPs) at channels C1, Cz, and C2. The data are shown separately for the four different combinations of greeble size (small vs. large) and vowels used in the greeble names (<i> vs. <a>). The analyzed N400 time window is highlighted in gray; time point 0 reflects the onset of the greeble's name.

**FIGURE 3:**
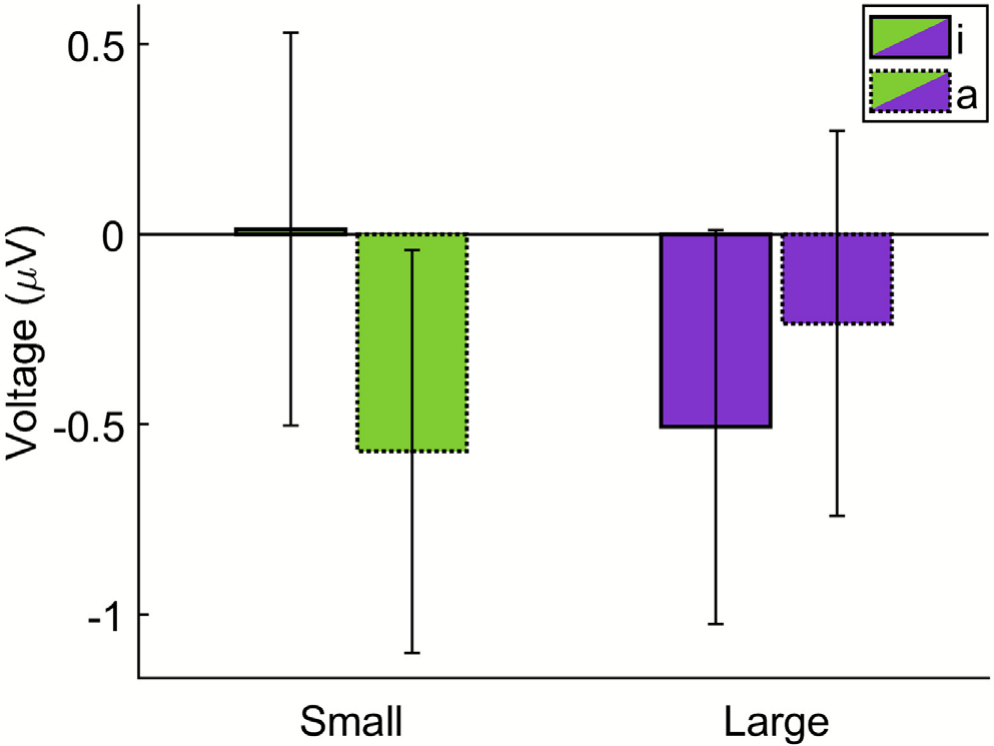
Mean N400 amplitudes for the four different combinations of greeble size (small vs. large) and vowels used in the greeble names (<i> vs. <a>). The data depict the average over channels C1, Cz, and C2. Error bars represent ±1 SEM.

We analyzed the N400 ERP component at nine central and parietal EEG channels within a time window of 448–498 ms after the onset of the greeble's name.^2^ The conducted four‐way ANOVA yielded no significant main effect of *greeble size*, *F*(1, 29) = 1.16, *p* = 0.290, $\eta_{p}^{2}$ = 0.039, or of *vowels*, *F*(1, 29) = 1.51, *p* = 0.229, $\eta_{p}^{2}$ = 0.049, and no significant interaction between *greeble size* and *vowels*, *F*(1, 29) = 3.66, *p* = 0.066, $\eta_{p}^{2}$ = 0.112. The interaction between *greeble size*, *vowels*, and *posteriority* was statistically significant, Greenhouse–Geisser‐adjusted *F*(1.27, 36.95) = 3.89, *p* = 0.047, $\eta_{p}^{2}$ = 0.118. Separate ANOVAs for the different levels of *posteriority* revealed a significant interaction between *greeble size* and *vowels* at the central EEG channels, *F*(1, 29) = 6.18, *p* = 0.019, $\eta_{p}^{2}$ = 0.176 (see Figure 2 for a visualization of the corresponding ERP curves; ERPs from all 64 EEG channels can be found in the article's Supporting Information). Here, the N400 amplitude was significantly less negative for names of the small greebles that contained the vowel letter <i> (*M* = 0.01 μV, SEM = 0.52 μV) compared to the vowel letter <a> (*M* = −0.57 μV, SEM = 0.53 μV), *t*(29) = 1.97, *p* = 0.029, *d*_*z*_ = 0.36. Regarding the large greebles, the comparison of the N400 amplitude between names with the vowel letter <a> (*M* = −0.24 μV, SEM = 0.51 μV) and names with the vowel letter <i> (*M* = −0.51 μV, SEM = 0.52 μV) was not significant, *t*(29) = 0.88, *p* = 0.194, *d*_*z*_ = 0.16 (see Figure 3).

**FIGURE 4:**
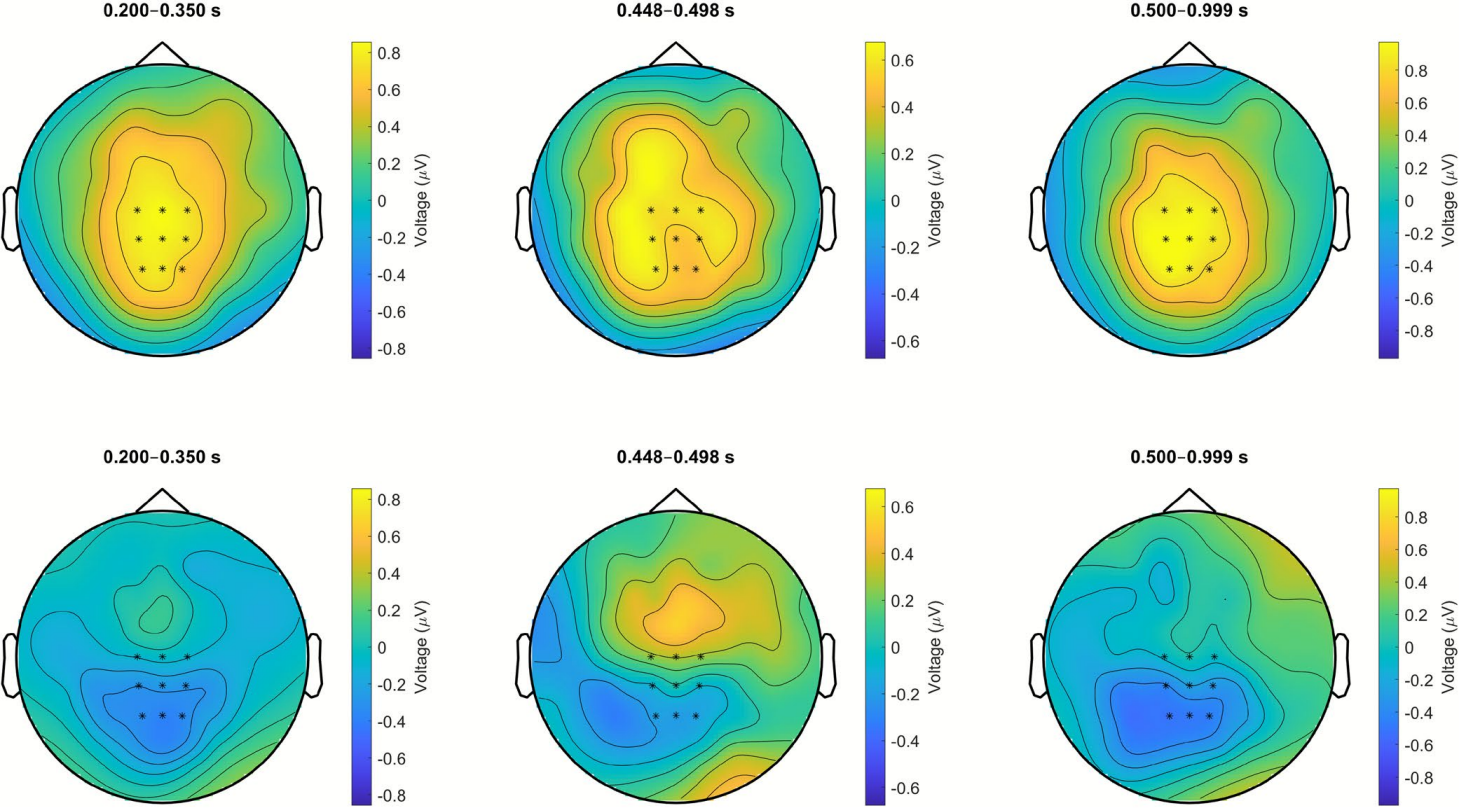
Topographic maps of the amplitude differences between the different vowel letters used in names of the small greebles (<i> − <a>; top row) and names of the large greebles (<a> − <i>; bottom row). Values are averaged within the three time windows that yielded significant effects in the statistical analyses. Analyzed electrodes are marked with an asterisk.

As visual inspection of the analyzed central and parietal EEG channels suggested potential differences between conditions in other time windows as well, we also performed exploratory analyses for these windows—noting, however, that this approach is associated with an increased risk of false positive results and should therefore be interpreted with caution (Luck and Gaspelin 2017). We divided the data into five additional time windows based on visual inspection of the individual signal deflections: 0–100 ms, 100–200 ms, 200–350 ms, 350–450 ms, and 500–999 ms. Except for a significant main effect of *vowels* in the third time window (200–350 ms), *F*(1, 29) = 15.77, *p* < 0.001, $\eta_{p}^{2}$ = 0.352 (<i>: *M* = 4.28 μV, SEM = 0.50 μV; <a>: *M* = 3.77 μV, SEM = 0.53 μV), the ANOVAs of the first four windows did not yield any significant main effects of *greeble size* or *vowels* and, importantly, no significant *greeble size* × *vowels* interaction, also not in combination with any of the spatial factors (all *p* > 0.05). Interestingly, regarding the fifth time window (500–999 ms), the data revealed significant main effects of both *greeble size*, *F*(1, 29) = 7.77, *p* = 0.009, $\eta_{p}^{2}$ = 0.211, and *vowels*, *F*(1, 29) = 13.52, *p* = 0.001, $\eta_{p}^{2}$ = 0.318, as well as a significant interaction effect between *greeble size* and *vowels*, *F*(1, 29) = 4.84, *p* = 0.036, $\eta_{p}^{2}$ = 0.143. Two‐tailed pairwise comparisons showed that the amplitude difference between the two vowel letters was significant for the small greebles, *t*(29) = 4.50, *p* < 0.001, *d*_*z*_ = 0.82 (<i>: *M* = 3.56 μV, SEM = 0.42 μV; <a>: *M* = 2.66 μV, SEM = 0.39 μV), but not for the large greebles, *t*(29) = 1.23, *p* = 0.227, *d*_*z*_ = 0.23 (<i>: *M* = 2.86 μV, SEM = 0.43 μV; <a>: *M* = 2.58 μV, SEM = 0.39 μV). Topographic difference maps for all time windows that yielded statistically significant effects are depicted in Figure 4.

## 3 Discussion

The present EEG study investigated the neural basis of size sound symbolism. We compared the processing of small‐sounding versus large‐sounding fictional names that were presented in reference to either small or large novel visual objects, namely greebles. In accordance with our hypotheses, the data revealed an effect of size congruency on the N400 ERP component, with sound‐symbolically congruent greeble–name pairings eliciting a reduced N400 amplitude at central electrode sites, in particular regarding the small greebles. Exploratory analyses demonstrated the presence of a congruency effect also during subsequent neural processing.

The present data are in line with a recent EEG study (Glim and Hillje 2025; see also Asano et al. 2015) that revealed a reduced N400 amplitude for sound‐symbolically congruent, compared to incongruent, car–name pairings, especially in terms of the car type cabriolet and fictional names that contained the vowel letter <i>—with both the cabriolet and <i> being related to properties such as smallness, thinness, lightness, and high speed. The study's results implied that the car's presentation led to a prediction‐based preactivation and thus facilitated neural processing of sound‐symbolically associated linguistic information. They did not clarify, however, to what extent individual sound‐symbolic dimensions such as size contributed to the overall effect and to what extent the effect was bound to stimulus categories such as cars that participants had already had plenty of real‐world experiences and associations with. By investigating pairings of novel visual objects and fictional names that were sound‐symbolically (in)congruent in terms of size, the present study extended the results and interpretation provided by Glim and Hillje (2025). We propose, in accordance with their argumentation and the extensive N400 literature (for a review, see Kutas and Federmeier 2011), that the context of the greeble entailed the generation of specific predictions about the upcoming greeble name—based on sound‐symbolic associations—and that these predictions then in turn allowed for a preactivation and thus facilitated neural processing and an easier integration of the matching, compared to mismatching, linguistic information.

Interestingly, pairwise comparisons in Glim and Hillje (2025) revealed the presence of such a neuro‐linguistic processing benefit for the cabriolet and names containing the vowel letter <i> but not for the car type SUV and names containing the vowel letter <o>. In the current study, we replaced <o> with <a> but still found a similar pattern of results, that is, a neural processing benefit for small greebles and names containing the vowel letter <i> but not for large greebles and names containing the vowel letter <a>. This similarity in N400 results suggests a systematic underlying difference between the different sound‐symbolic associations, with <i> potentially being more strongly associated with physical properties such as size than <o> and <a>. A comparable conclusion was also reached by Blasi et al. (2016), who analyzed a broad sample of existing languages and found a robust association between smallness and the vowel <i> but no equivalent pattern for largeness. In line with this finding, Winter and Perlman (2021) reported that <i> exhibited the highest predictive performance among phonemes of English size adjectives. Beyond size, <i> has also been shown to produce asymmetric effects in other sound‐symbolic domains such as valence (Schmidtke et al. 2025). The full extent and precise causes of these and similar asymmetries in sound‐symbolic associations remain to be fully understood and are therefore recommended to be targeted in more detail by future theoretical and experimental investigations.

In contrast to Glim and Hillje (2025), the N400 congruency effect in the present study was significant at central rather than parietal electrode sites (with the topographic map indicating an expansion towards left and frontal sites). This finding is in line with the N400 data of Asano et al. (2015), who likewise observed an effect over central brain regions. All three findings fall within the general topographic range of the N400 component, which exhibits a typical maximum at centro‐parietal channels (see e.g., Kutas and Federmeier 2011). It is well known that the N400's precise topography can vary as a function of stimulus properties (e.g., Caldara et al. 2004; Holcomb et al. 1999; Kounios and Holcomb 1994; Schöne et al. 2018), and one might thus speculate that the observed spatial variation is related to the different types of sound‐symbolic stimuli employed. In particular, it stands out that the car concepts presented in Glim and Hillje (2025) were semantically richer and presumably activated a broader set of perceptual and conceptual associations than the bouba/kiki‐like shapes of Asano et al. (2015) and the greebles used here, both of which were artificially created, unfamiliar stimulus categories. Importantly, though, the currently available data do not allow for definite conclusions regarding potential spatial processing differences between different types of sound‐symbolic associations, and future endeavors are encouraged—based on the present findings—to directly manipulate stimulus types within experimental studies in order to gain a deeper understanding of this matter.

Besides the hypothesized size congruency effect on the N400 ERP component, we observed differences between conditions in other time windows as well. First, the data revealed a significant main effect of *vowels* in the time range of 200–350 ms, which, based on visual inspection of the ERP curves, corresponded to the P200 component. This component is part of the standard response to visual stimuli and presumably reflects aspects of attention‐related higher‐order perceptual processing (e.g., feature detection; Luck and Hillyard 1994). Its amplitude varies with word frequency (Dambacher et al. 2006) and the frequency of the first phonological syllable in pseudowords (Kwon et al. 2011), with the visual similarity between words (Kong et al. 2012), the recognition of spelling errors in words (Larionova and Martynova 2022), and the number of prevalent semantic associations (Stuellein et al. 2016), among other variables. Apart from manipulating the letters <i> versus <a>, pseudowords in the present study were held constant between conditions, which went along with potential differences in a number of other perceptual and linguistic features (e.g., frequency) that might have contributed to the observed P200 difference. A controlled manipulation of these features would be required to map their individual influence on the P200's amplitude. Crucially, however, (size‐unrelated) systematic differences between pseudoword conditions manifest in a main effect of *vowels*, as seen here, but do not explain the sound‐symbolic interaction effect that is central to the present investigation.

It might therefore be of greater interest that the exploratory analyses revealed such an interaction effect also in a late, post‐N400 time window, 500–999 ms after greeble name onset. Visual inspection suggested that this time window was dominated by a late positive component (LPC). The emergence of a late positivity in the signal is typically thought to indicate human memory functions, with the deflection's amplitude presumably reflecting the extent of recollective processing elicited by a given input stimulus (e.g., Paller et al. 1995; Rugg et al. 1995, 1996; Tsivilis et al. 2015). It might thus be speculated that the increased amplitude observed here for the combination of small object and vowel <i>, compared to <a>,^3^ is indicative of a stronger or more elaborate representation of the respective association in memory. This interpretation of the present LPC finding is in line with the fact that the association between smallness and high acoustic frequencies/<i> is prevalent both in the physical world and a multitude of existing languages (Blasi et al. 2016; Ohala 1995; see also Bakker et al. 2015, who reported a more positive LPC for novel words that were preceded by semantically associated primes). Interestingly, though, it might be noted that LPC effects are typically observed in the context of decision‐relevant memory signals (e.g., Yang et al. 2019), raising the question of whether and how conscious access (see also Rohaut et al. 2015) and intention played a role in the present findings. While the LPC is typically linked to explicit memory, there is also evidence of LPC effects during incidental tasks that do not require explicit memory judgments (Griffin et al. 2013; Li et al. 2015, 2016). Griffin et al. (2013), for example, reported an LPC during incidental stimulus encoding that was predictive of later memory performance, potentially because (in this case uninstructed) recollective processing during the encoding phase served to improve memory functions (a phenomenon known as the testing effect; e.g., Roediger III and Karpicke 2006). Regarding size sound symbolism, Preziosi and Coane (2017) reported better recall from associative memory for sound‐symbolically congruent, compared to incongruent, stimulus pairings. Although we did not assess memory performance, it is conceivable that modifications of memory traces were also at play in the present study, presumably on an implicit rather than explicit level (see below). Yet, more targeted examinations of sound‐symbolic effects on the LPC—and other memory‐related ERP components as well as relevant behavioral correlates—are undoubtedly required for final conclusions on this matter.

Importantly, the sound‐symbolic effects in the present study were found in the absence of respective task demands such as an explicit matching task, associated behavioral responses, and participants' conscious awareness of the sound‐symbolic manipulation, with the latter confirmed by a postexperimental survey item and during debriefing. The present results are thus consistent with an automatic (unconscious and task‐irrelevant) activation of sound‐symbolic associations during external stimulus processing, in this case of the greeble object. This conclusion is in line with behavioral data from Parise and Spence (2012), who observed a sound‐symbolic congruency effect across the entire spectrum of response times in an IAT. The authors argued that the fastest responses were primarily driven by automatic processes, whereas the slower responses presumably reflected a mixture of automatic processes and cognitive strategies. In contrast to Parise and Spence (2012), it appears unlikely that explicit cognitive strategies played a role in the present study, as the task employed here via the attention check questions required participants to focus on remembering the greeble's position as well as the consonants within the greeble's name—both of which were unrelated to the sound‐symbolic manipulation under investigation. Provided that attention is sufficiently captured without being drawn to the critical manipulation, the questions' exact nature should be of minor relevance. Future studies are encouraged to vary the type of attention‐capturing material to confirm this assumption.

Speaking of the stimulus material, we would also like to address the fact that the linguistic information in the present study was presented visually (see also Glim and Hillje 2025), as compared to auditorily (see Asano et al. 2015, who examined non‐reading infants). There is broad consensus that phonological codes are automatically activated during word reading in skilled readers (e.g., Braun et al. 2009, 2015; Coltheart et al. 1994; Rayner et al. 1995). Yet, in addition to phonological information, written (pseudo)words also entail orthographic information/grapheme‐related visual cues that might contribute to effects of sound symbolism. Thompson and Estes (2011) noted a correlation between the number of large‐sounding phonemes in the nonwords used in their experiment and the nonwords' width in pixels. This relationship, however, was deemed unlikely to fully explain their findings on size sound symbolism, and a subsequent experiment using auditory presentation yielded largely corresponding results. Future studies might be interested in conducting a similar comparison with regard to the present data. In addition, future studies are encouraged to vary the pattern used for visual or auditory pseudoword creation to further test the robustness of the present findings, for example by manipulating the number of large‐ or small‐sounding vowels in the pseudowords or by contrasting consonants instead, while ideally controlling for as many other differences between pseudowords as possible. Finally, we also welcome direct replications of our study as well as the expansion of EEG research into additional domains of sound symbolism, such as valence, to establish a more comprehensive and biologically grounded understanding of this intriguing phenomenon.

In conclusion, the present study adds neurophysiological evidence to the idea that physical properties such as size are intrinsically linked to specific linguistic features. It demonstrates the impact of such sound‐symbolic associations on the brain's information processing system, and it thereby suggests that size sound symbolism, in particular regarding smallness and the vowel <i>, might constitute an inherent component of how we naturally experience the physical and linguistic world around us.

## Author Contributions

**Sarah Glim:** conceptualization, formal analysis, funding acquisition, investigation, software, methodology, visualization, writing – original draft, writing – review and editing. **Ralf Rummer:** writing – review and editing.

## Funding

This work was supported by the Central Research Fund (ZFF) of the University of Kassel.

## Conflicts of Interest

The authors declare no conflicts of interest.

## Supporting information


**Data S1:** psyp70190‐sup‐0001‐Supinfo.zip.

## Data Availability

The data that support the findings of this study are available from the corresponding author upon reasonable request.

## Endnotes

^1^
Pseudoword creation was coarsely based on Thompson and Estes (2011; Experiment 1), with the main deviations being that we used the pattern CVCVC instead of CVCVCV, focused on contrasting the individual vowel letters <i> and <a> rather than a mixture of vowels, and kept consonants constant between conditions.

^2^
We note that there is considerable variability in the literature when it comes to selecting the EEG channels and time window for N400 analyses (Šoškić et al. 2022). Except for a smaller time window (±25 ms rather than ±50 ms, based on the visible component's form), the present analysis was identical to that of Glim and Hillje (2025). Running the analysis with the larger window yielded qualitatively identical results, with the exception that the interaction between *greeble size*, *vowels*, and *posteriority* was only marginally significant (Greenhouse–Geisser‐adjusted *p* = 0.054). Qualitatively identical results were also obtained when determining the N400's trough by averaging the signal only over the nine analyzed central and parietal EEG channels instead of all EEG channels, which yielded an analysis window of 451–501 ms after the onset of the greeble's name.

^3^
The directionality of this effect is identical to that found for the N400 component. Based on the outlined theoretical considerations, visual inspection of the ERP curves, and differences in topographic distributions, we consider an interpretation of the two effects in terms of two distinct neural processes, rather than one prolonged mechanism, the more plausible account.
