# Supplementary material for: Size Sound Symbolism Modulates Linguistic Processing: An ERP Study
Source: Psychophysiology. 2025 Nov 28;62(12):e70190. doi: 10.1111/psyp.70190 (PMC12662948; doi:10.1111/psyp.70190)

Grand-Average ERPs for all EEG Channels

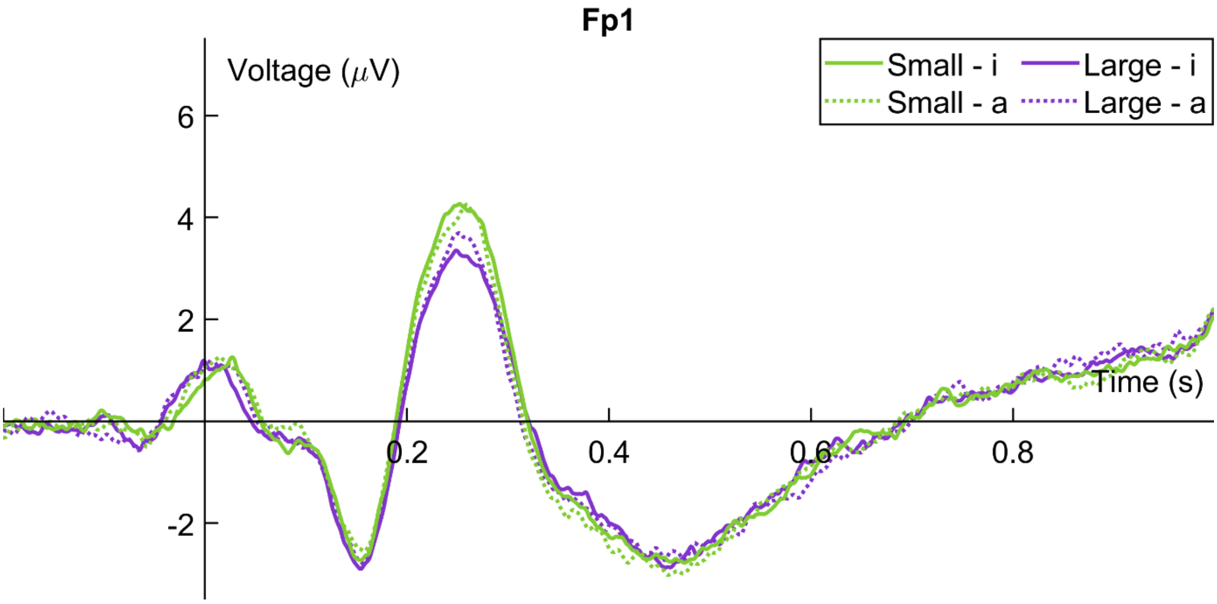

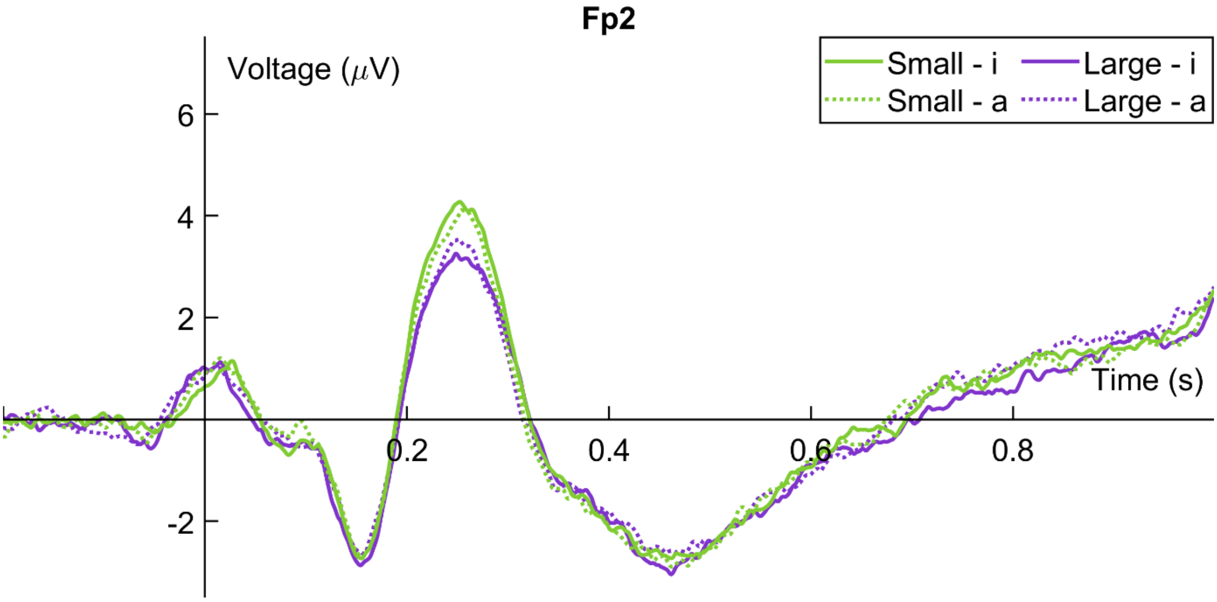

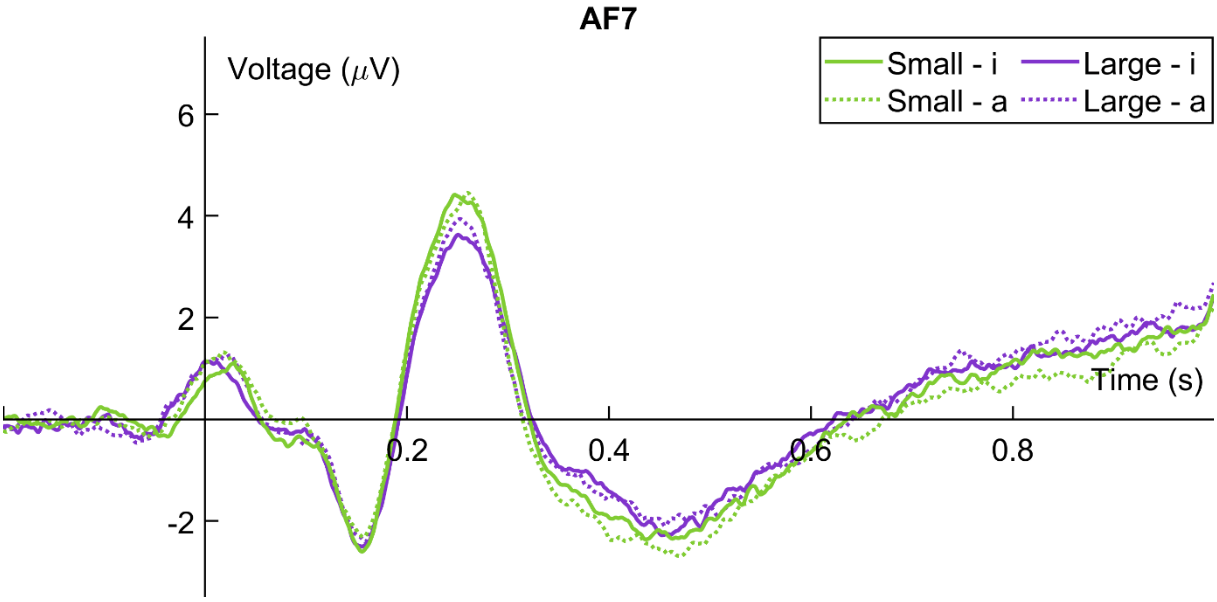

Grand-Average ERPs for all EEG Channels

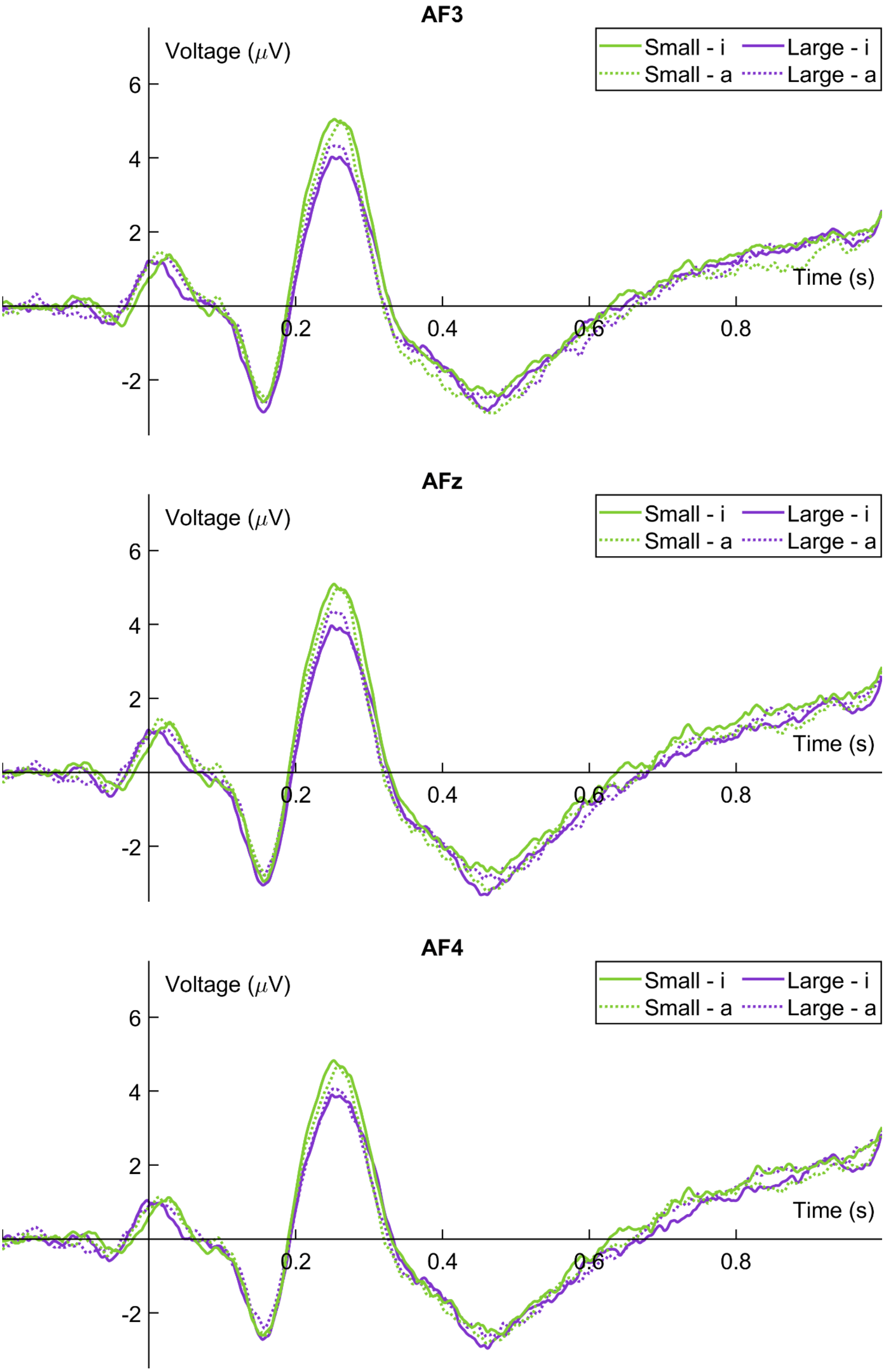

Grand-Average ERPs for all EEG Channels

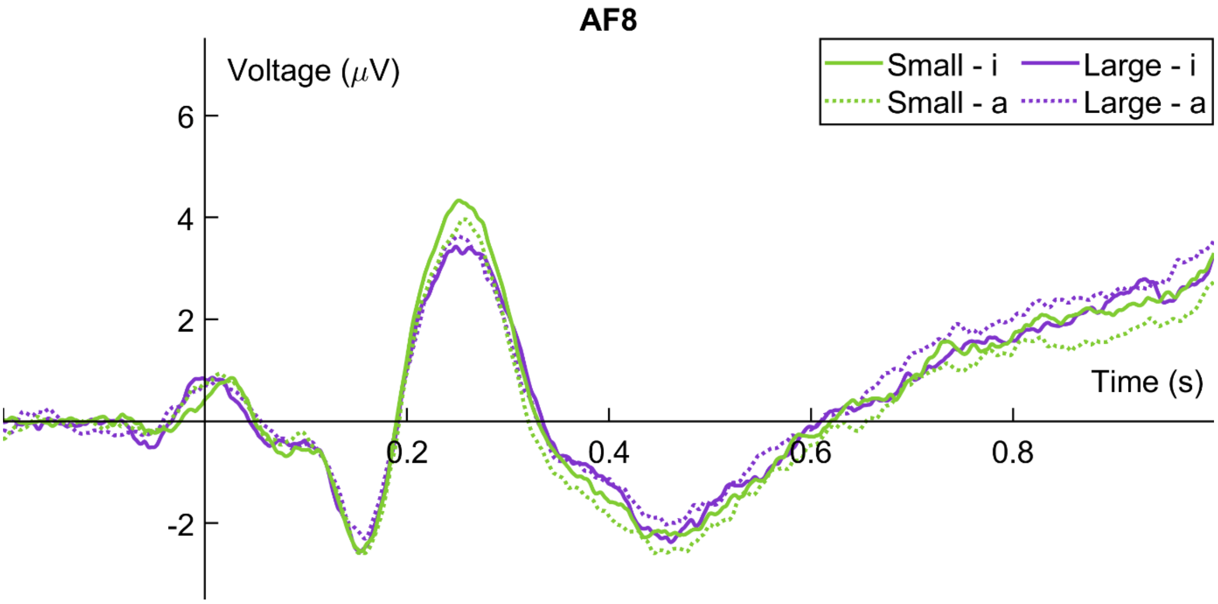

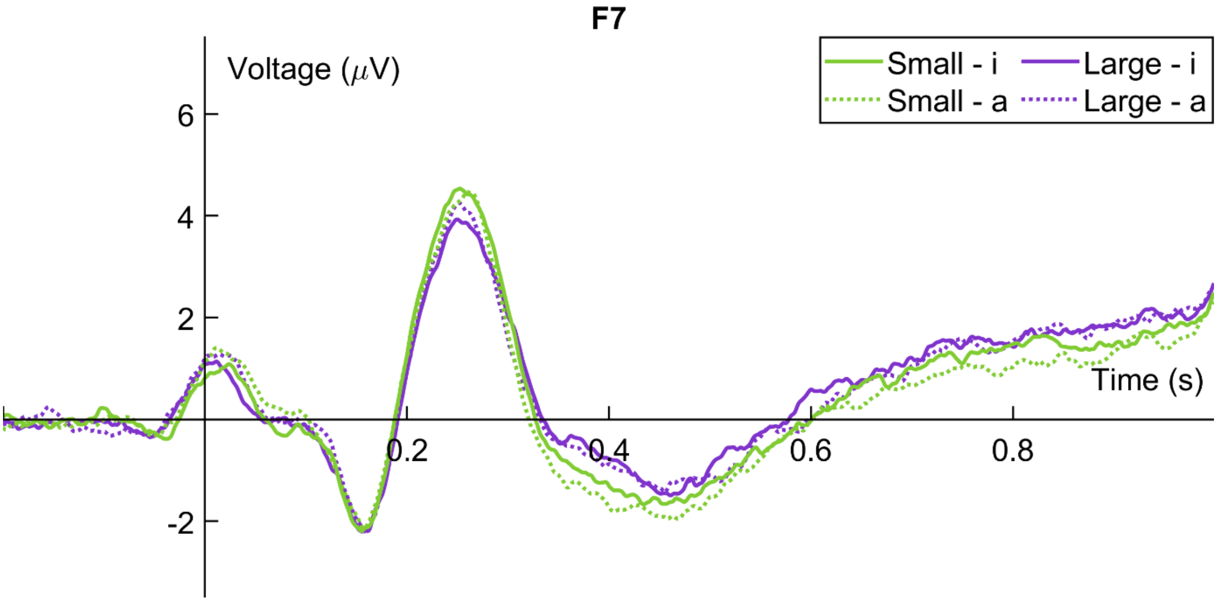

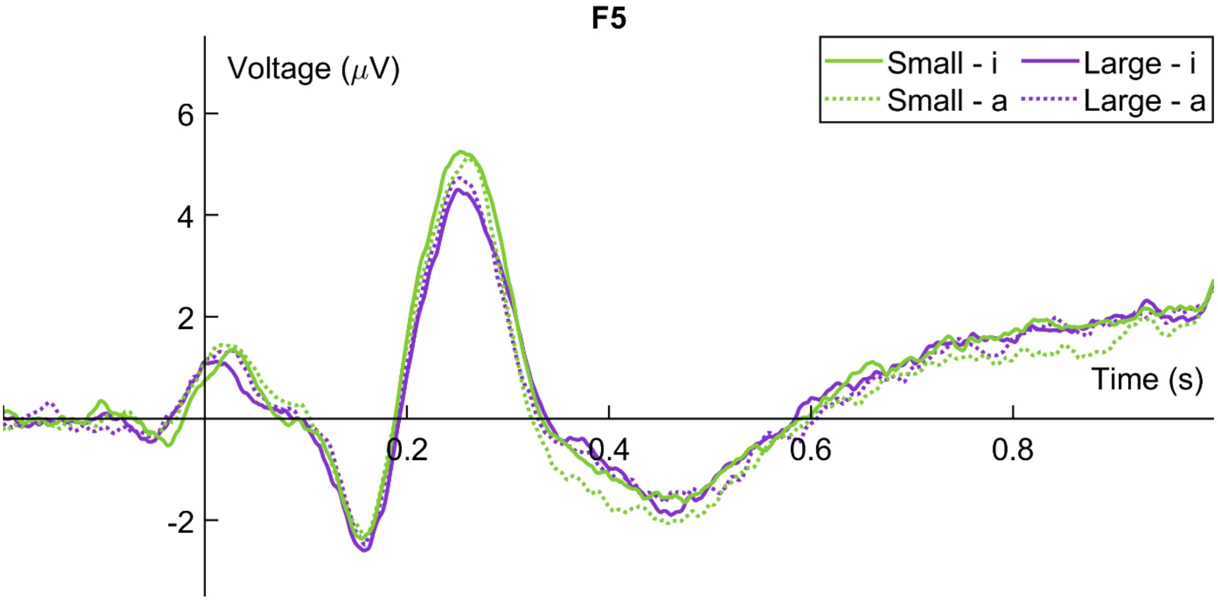

Grand-Average ERPs for all EEG Channels

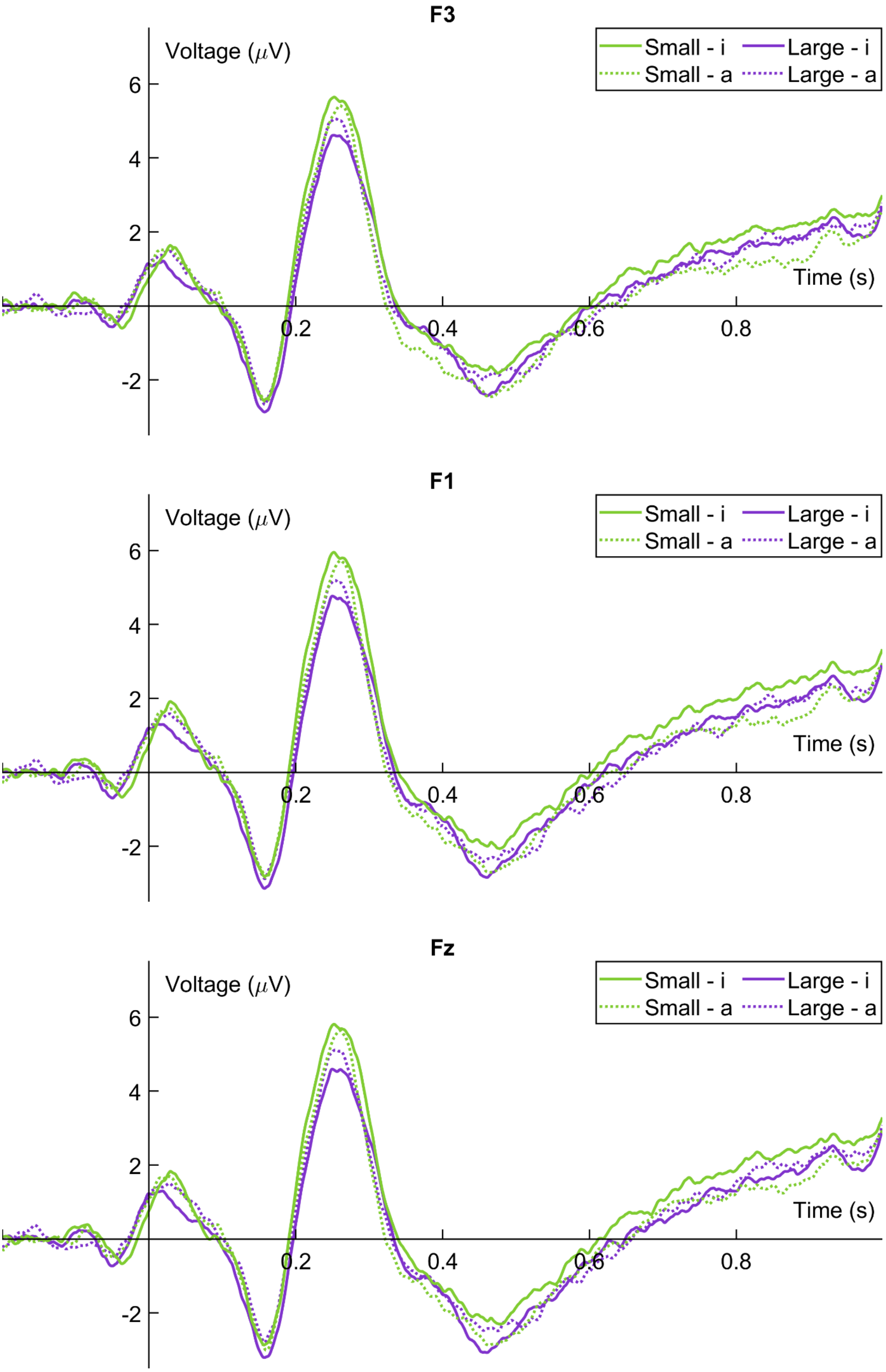

Grand-Average ERPs for all EEG Channels

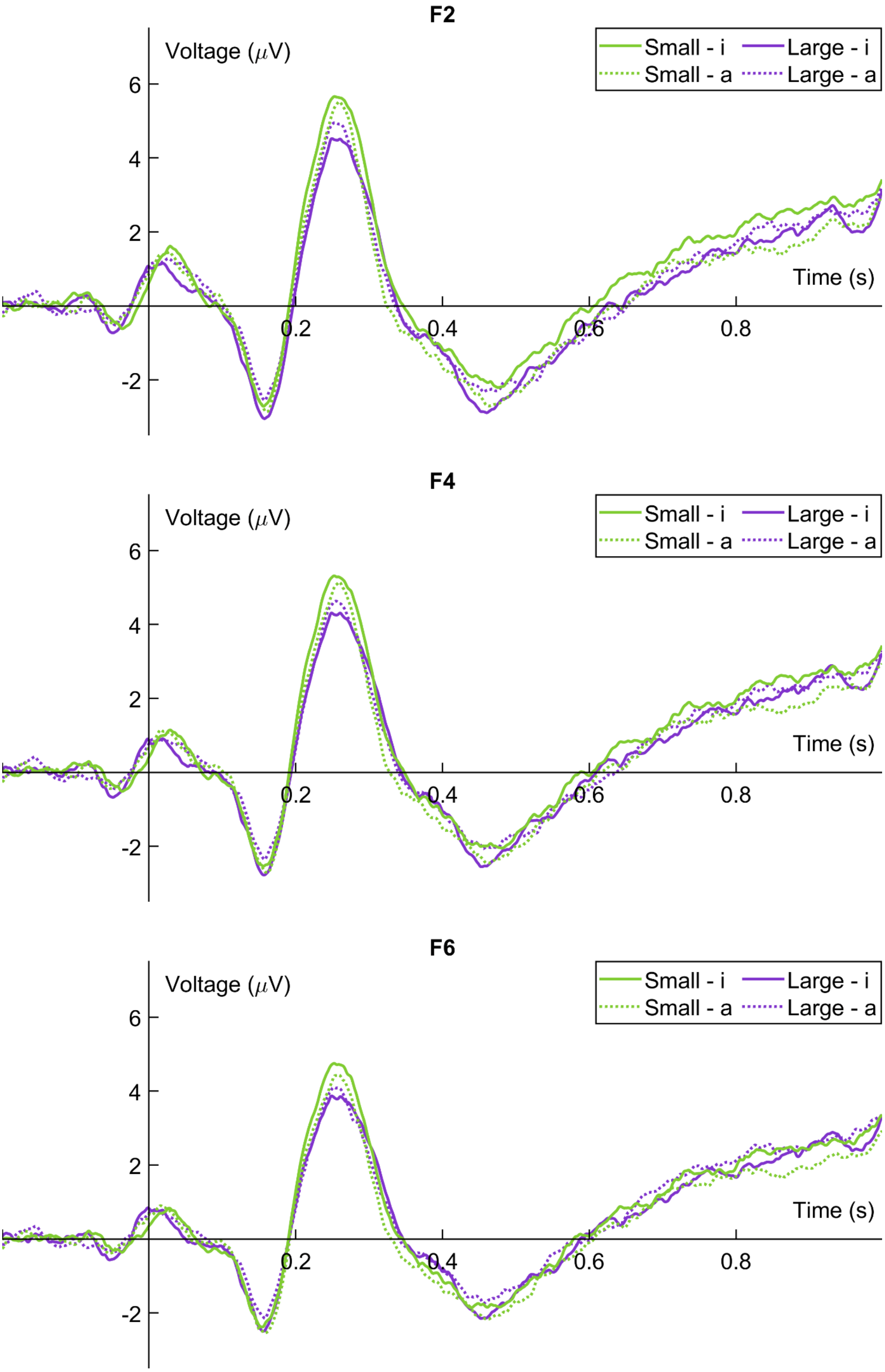

Grand-Average ERPs for all EEG Channels

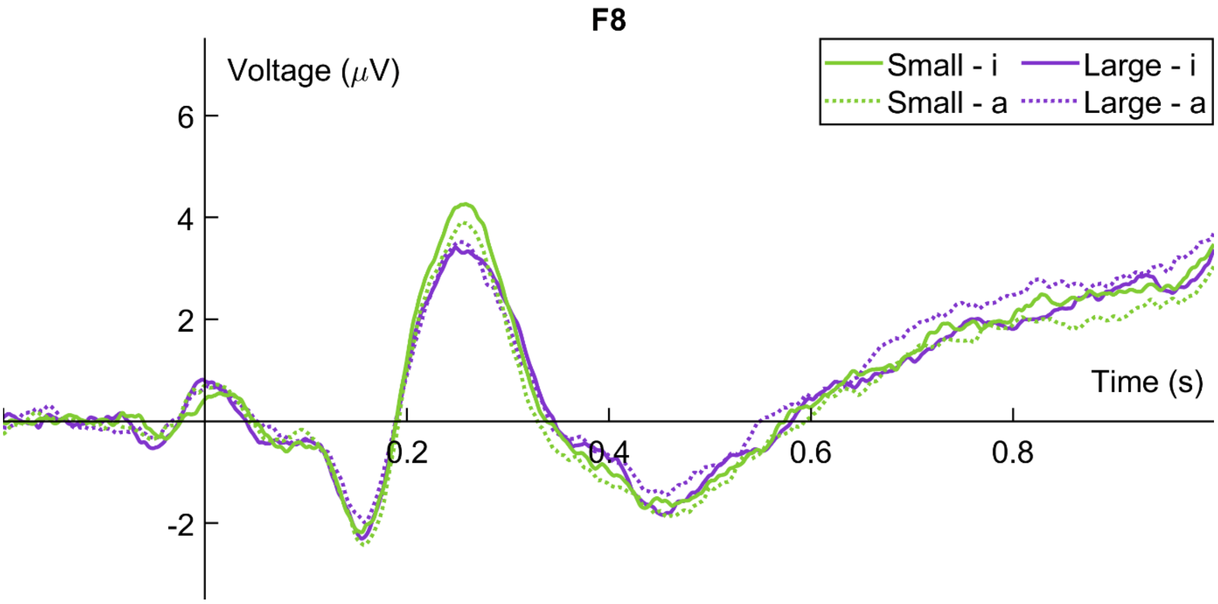

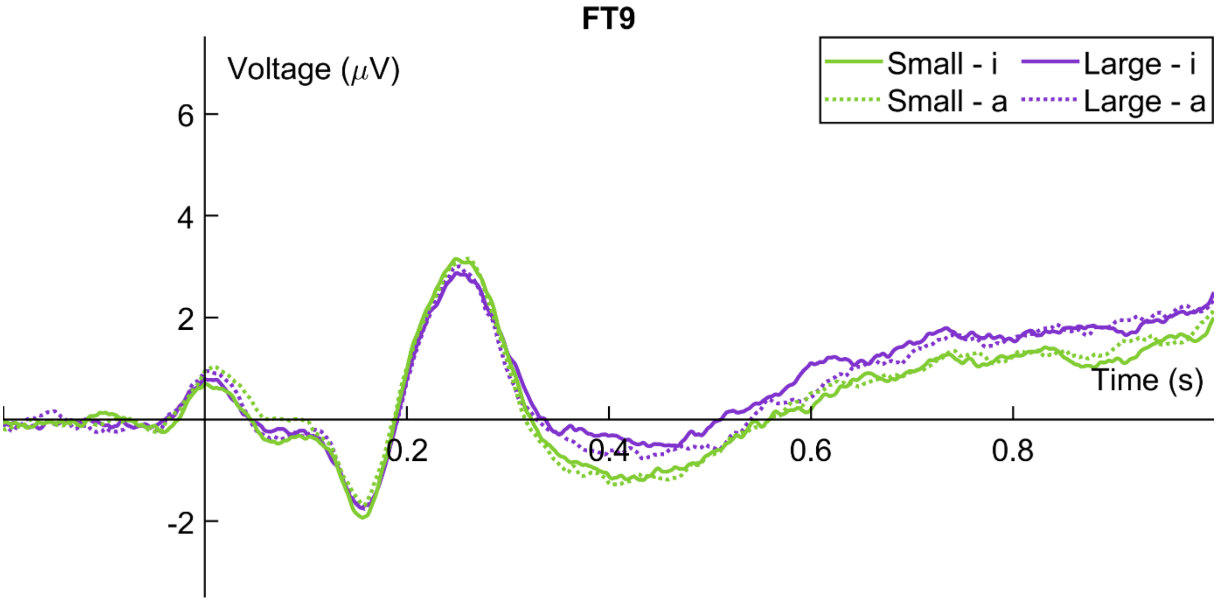

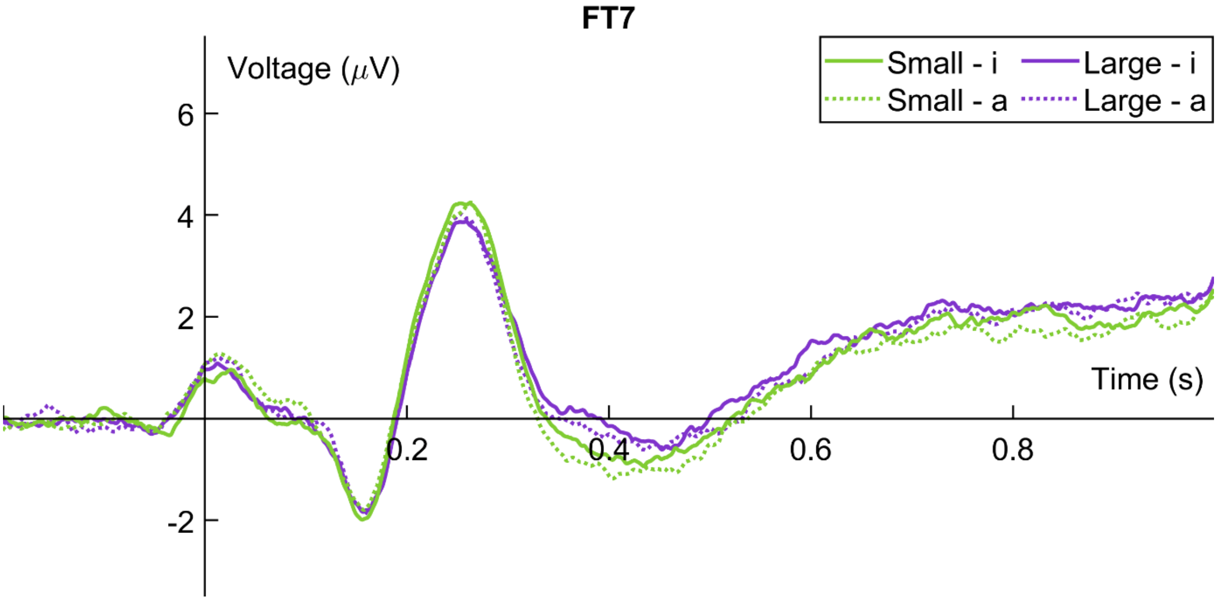

Grand-Average ERPs for all EEG Channels

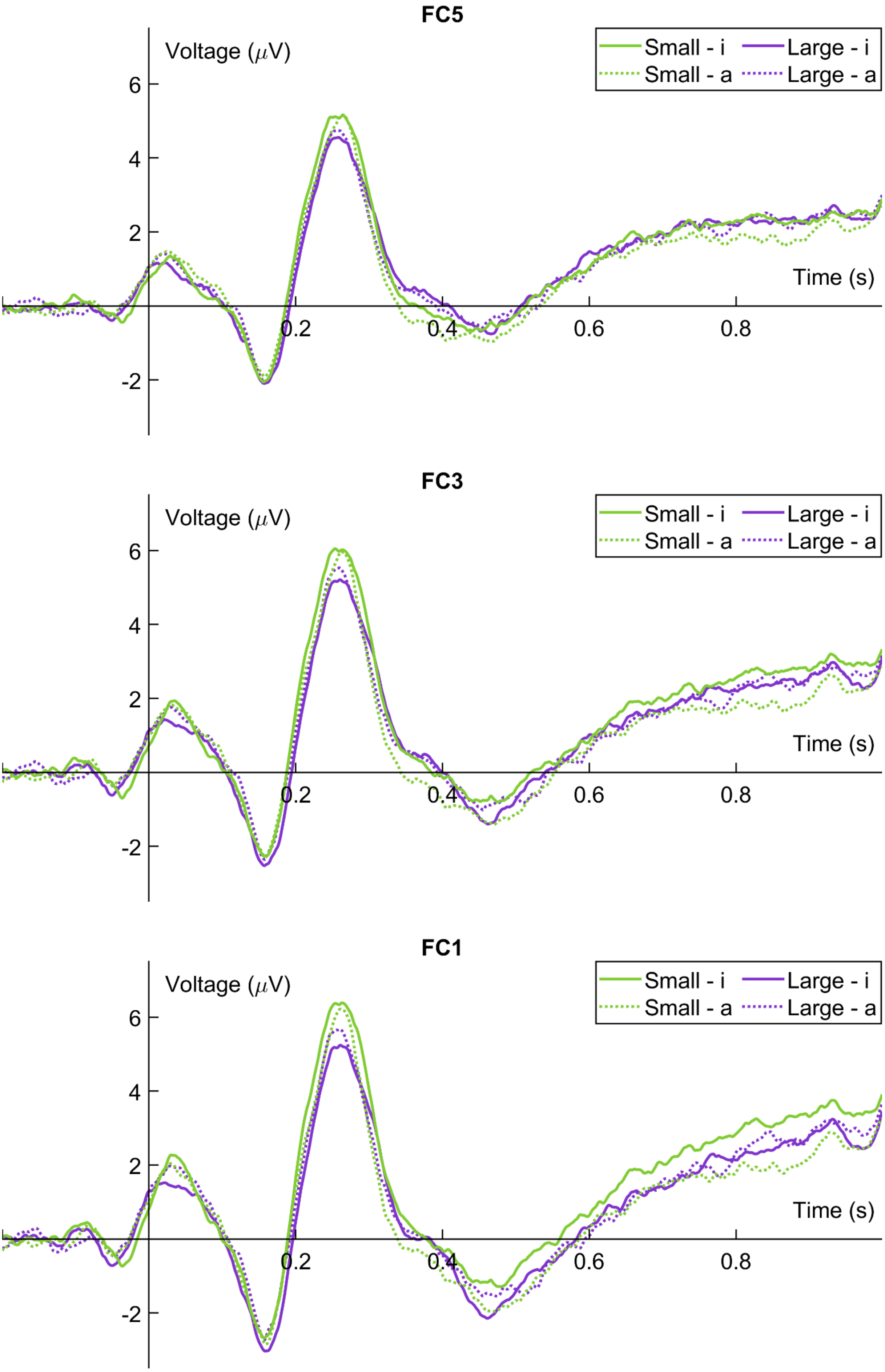

Grand-Average ERPs for all EEG Channels

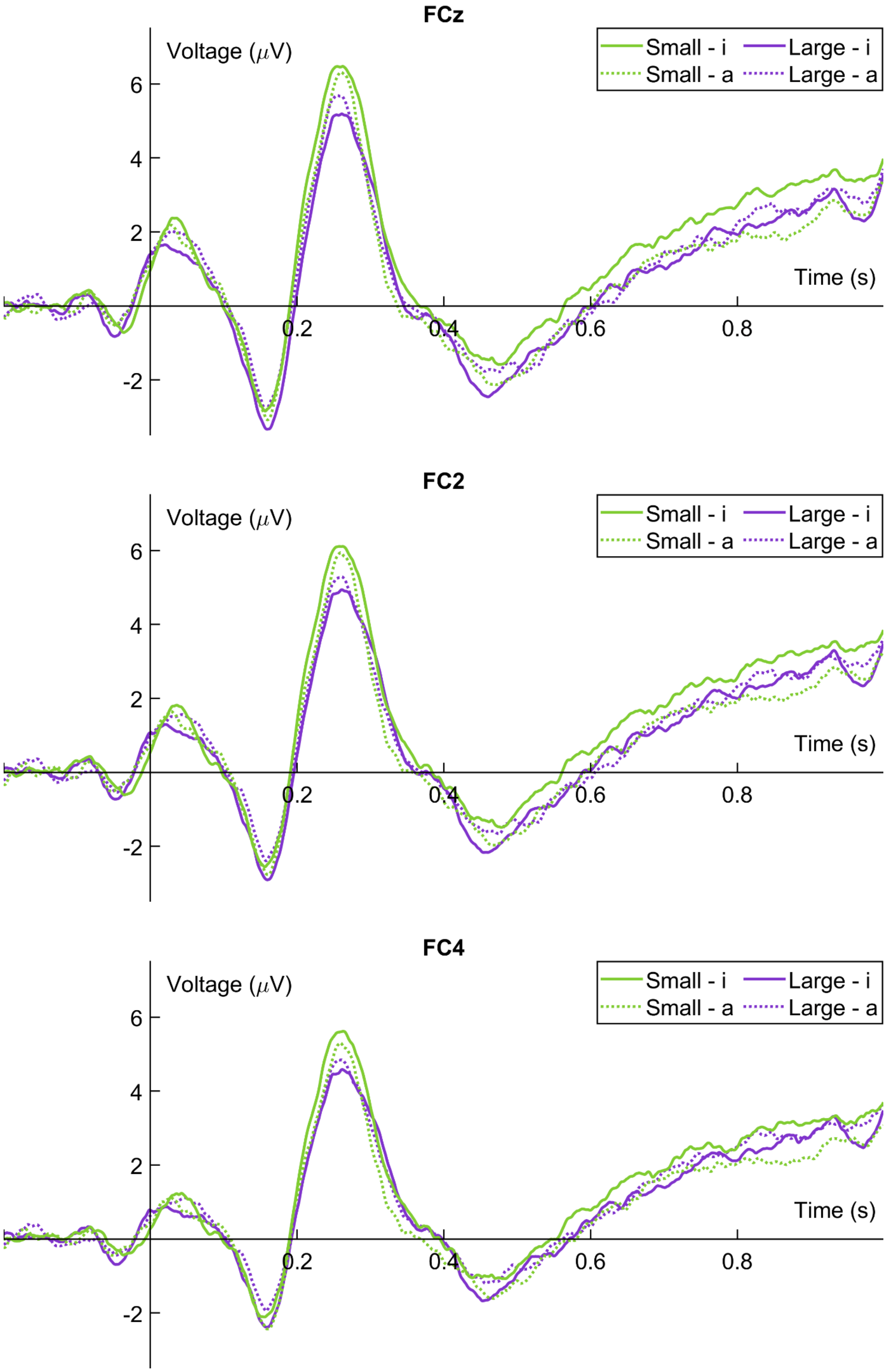

Grand-Average ERPs for all EEG Channels

FC6

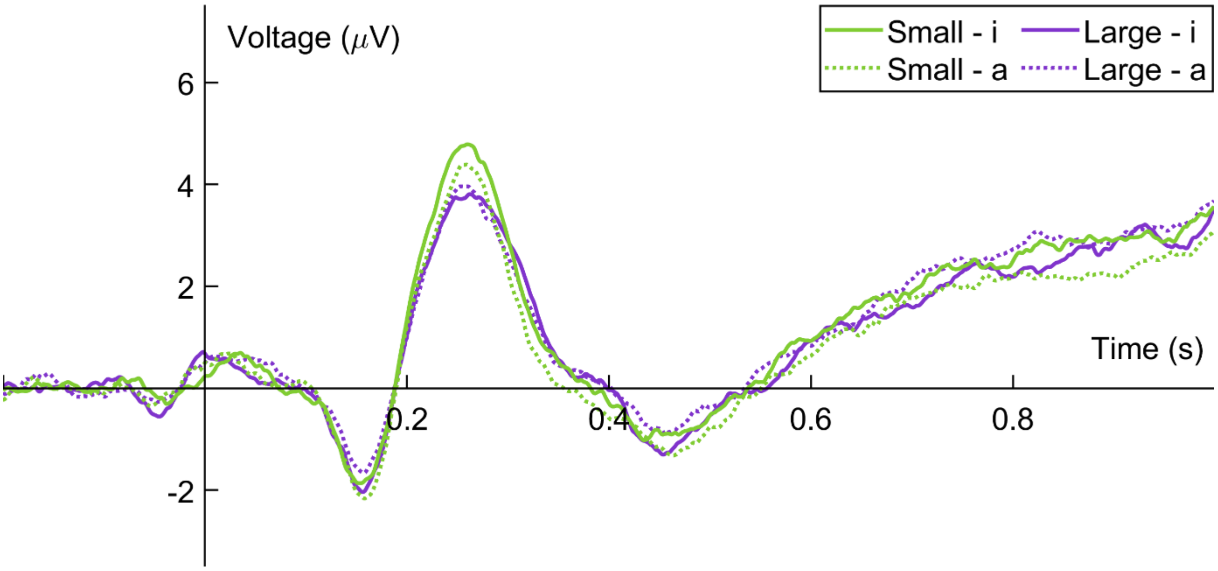

FT8

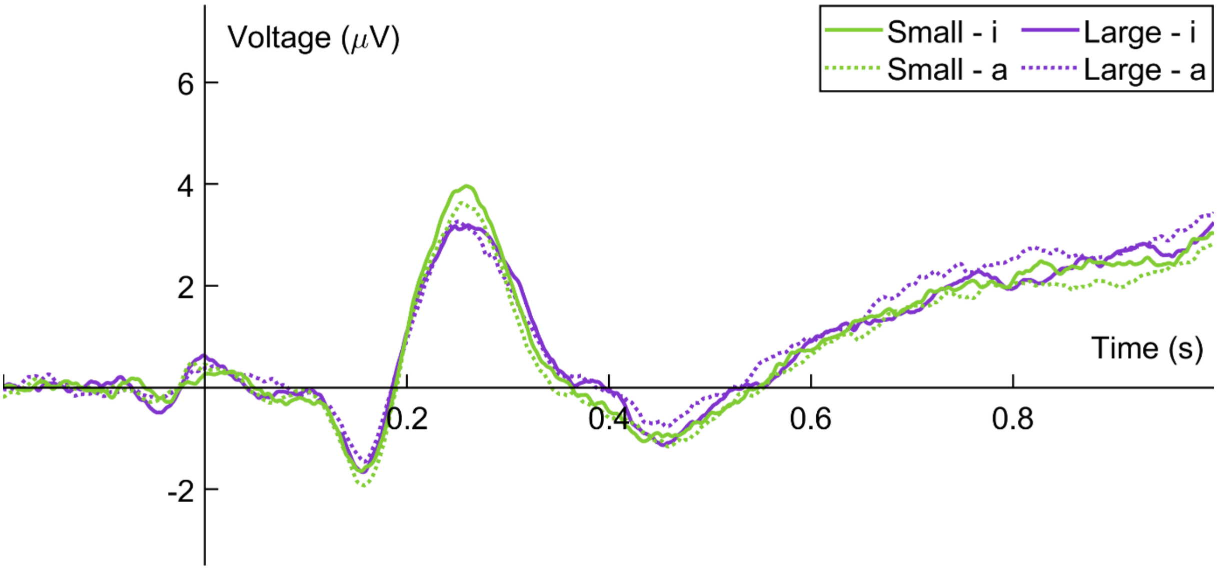

FT10

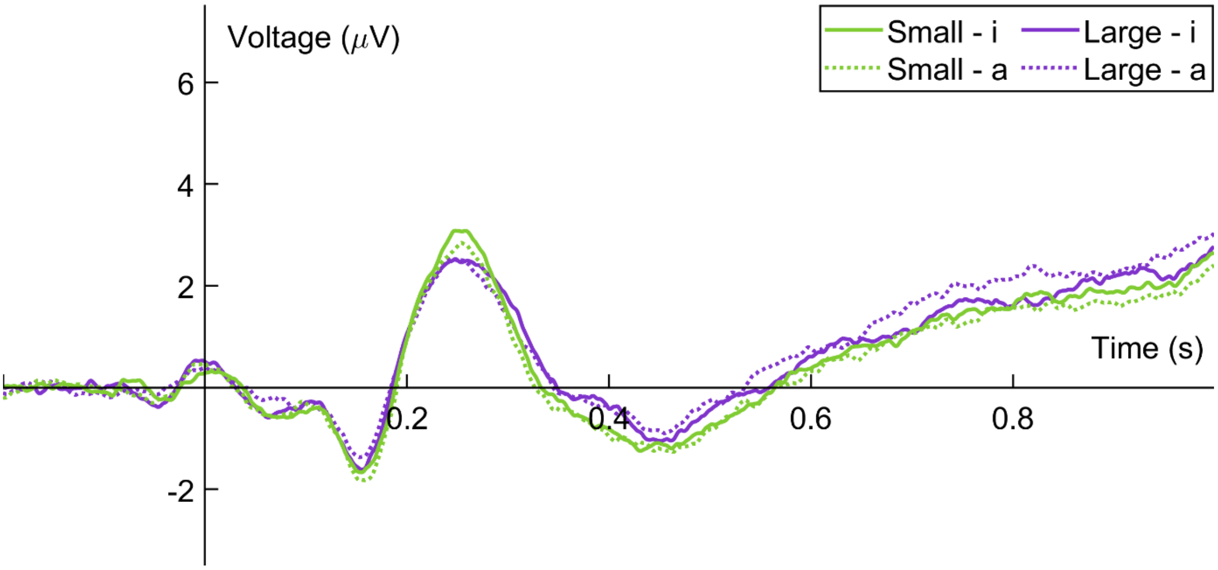

Grand-Average ERPs for all EEG Channels

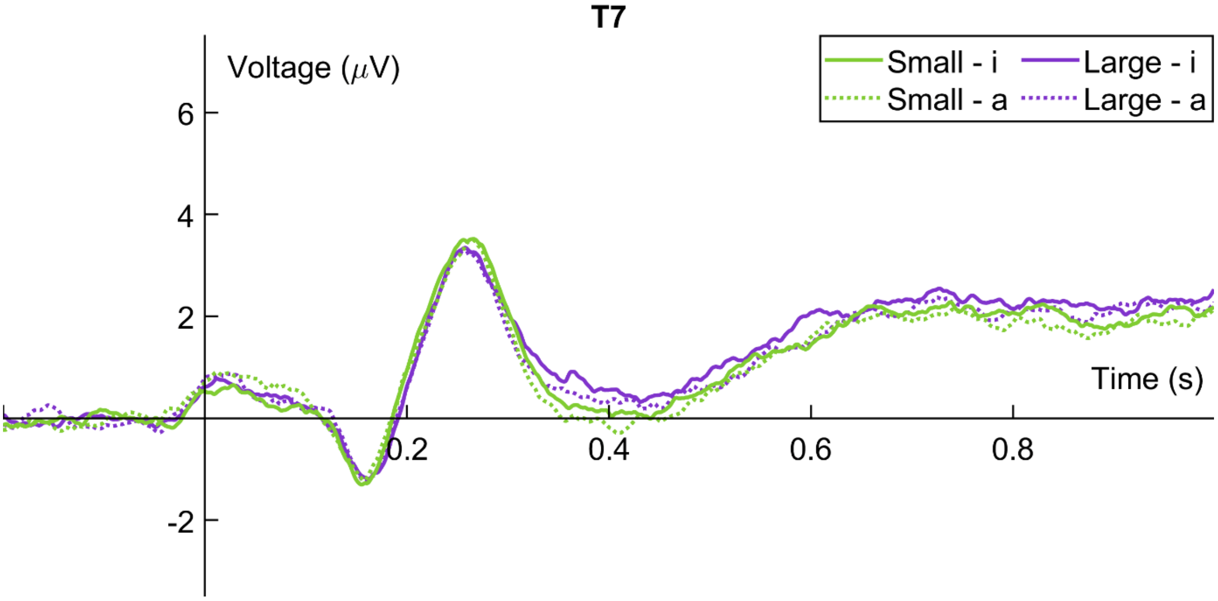

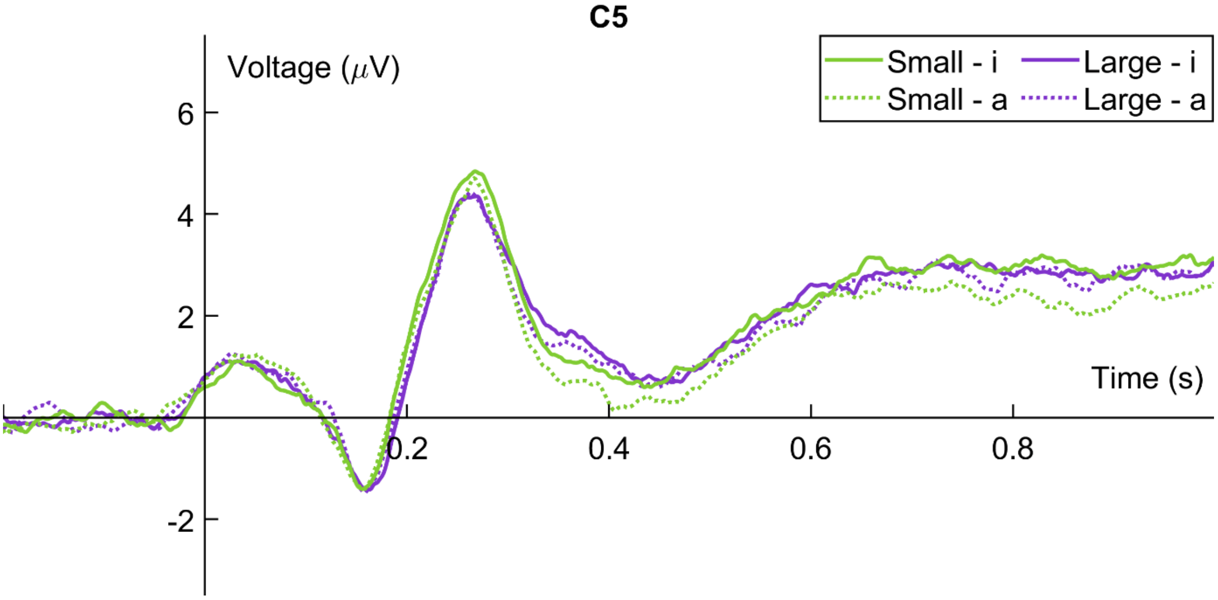

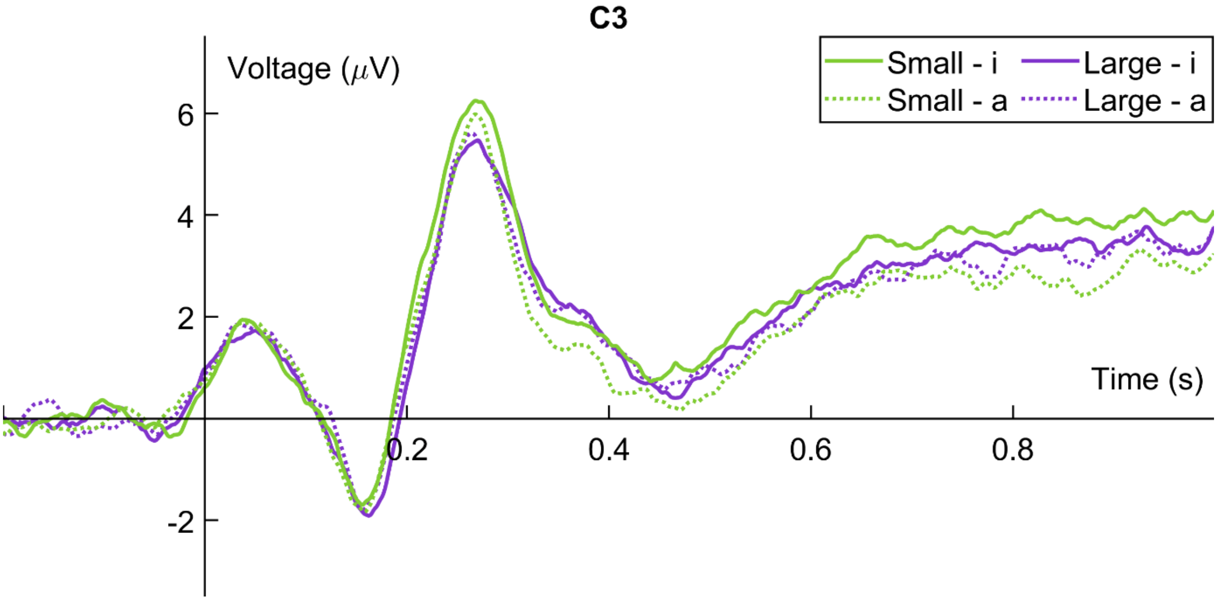

Grand-Average ERPs for all EEG Channels

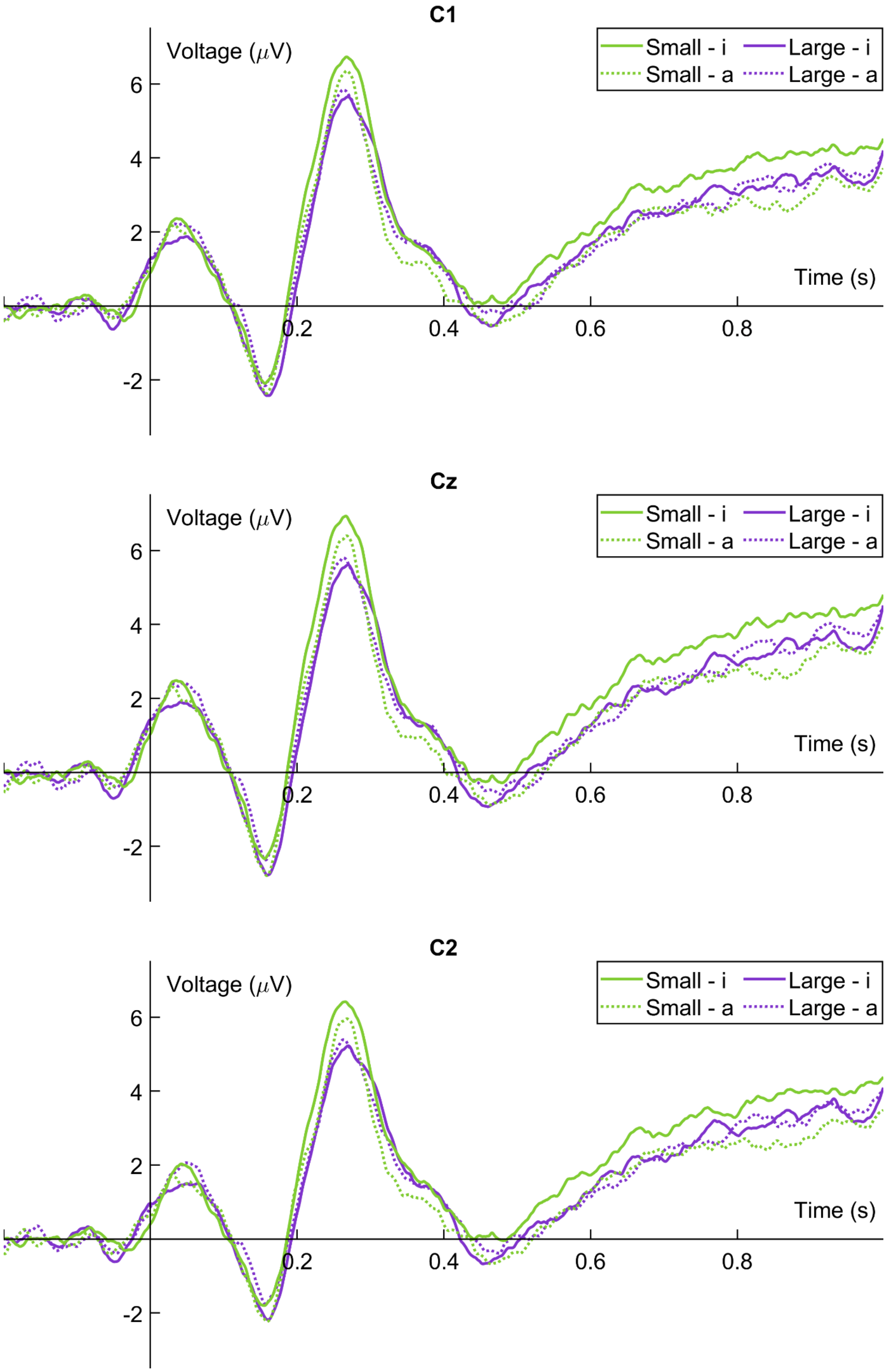

Grand-Average ERPs for all EEG Channels

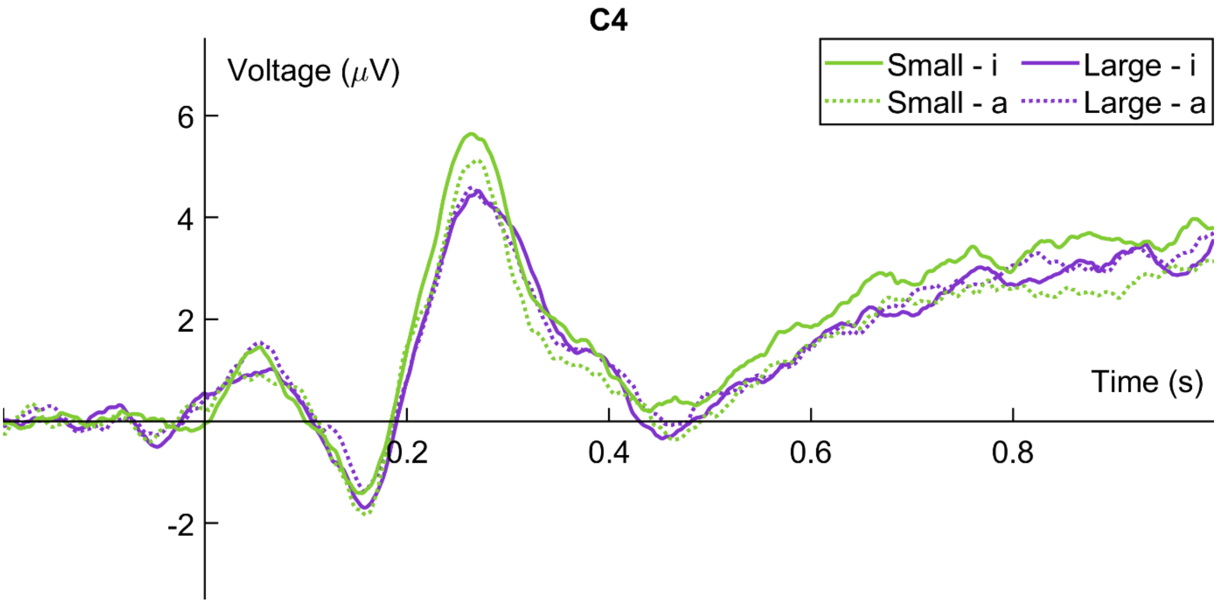

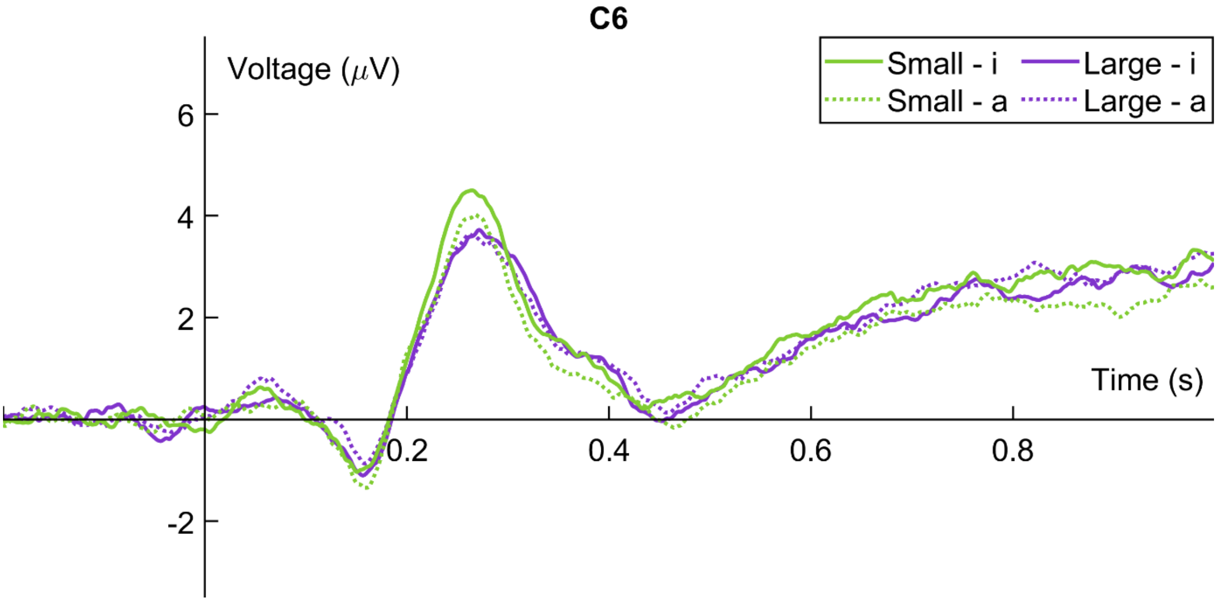

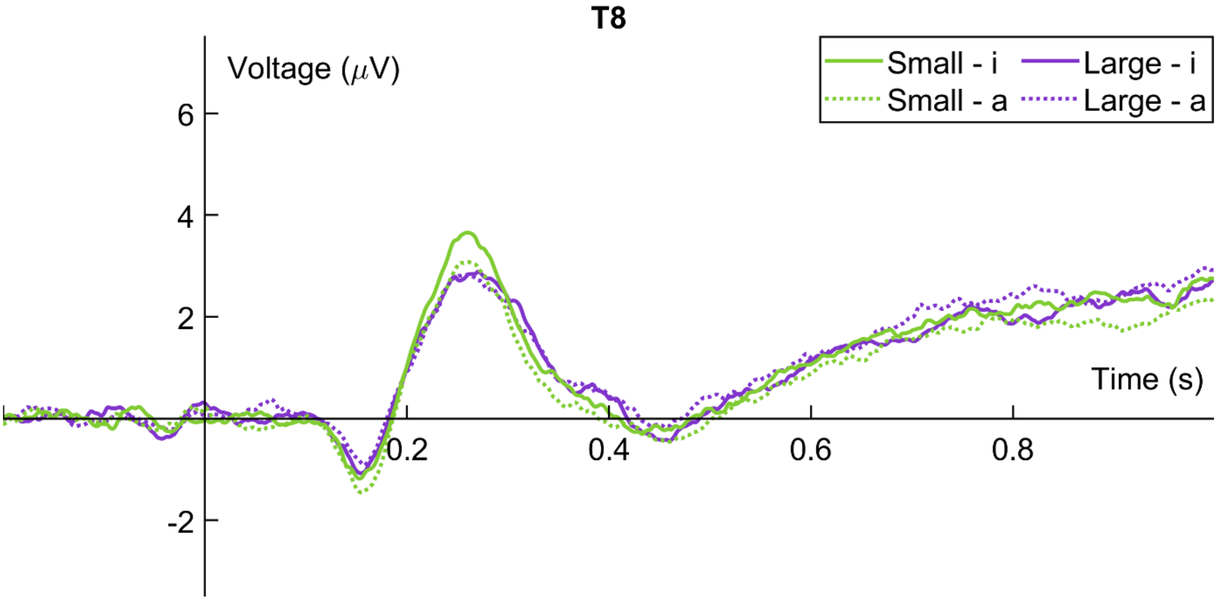

Grand-Average ERPs for all EEG Channels

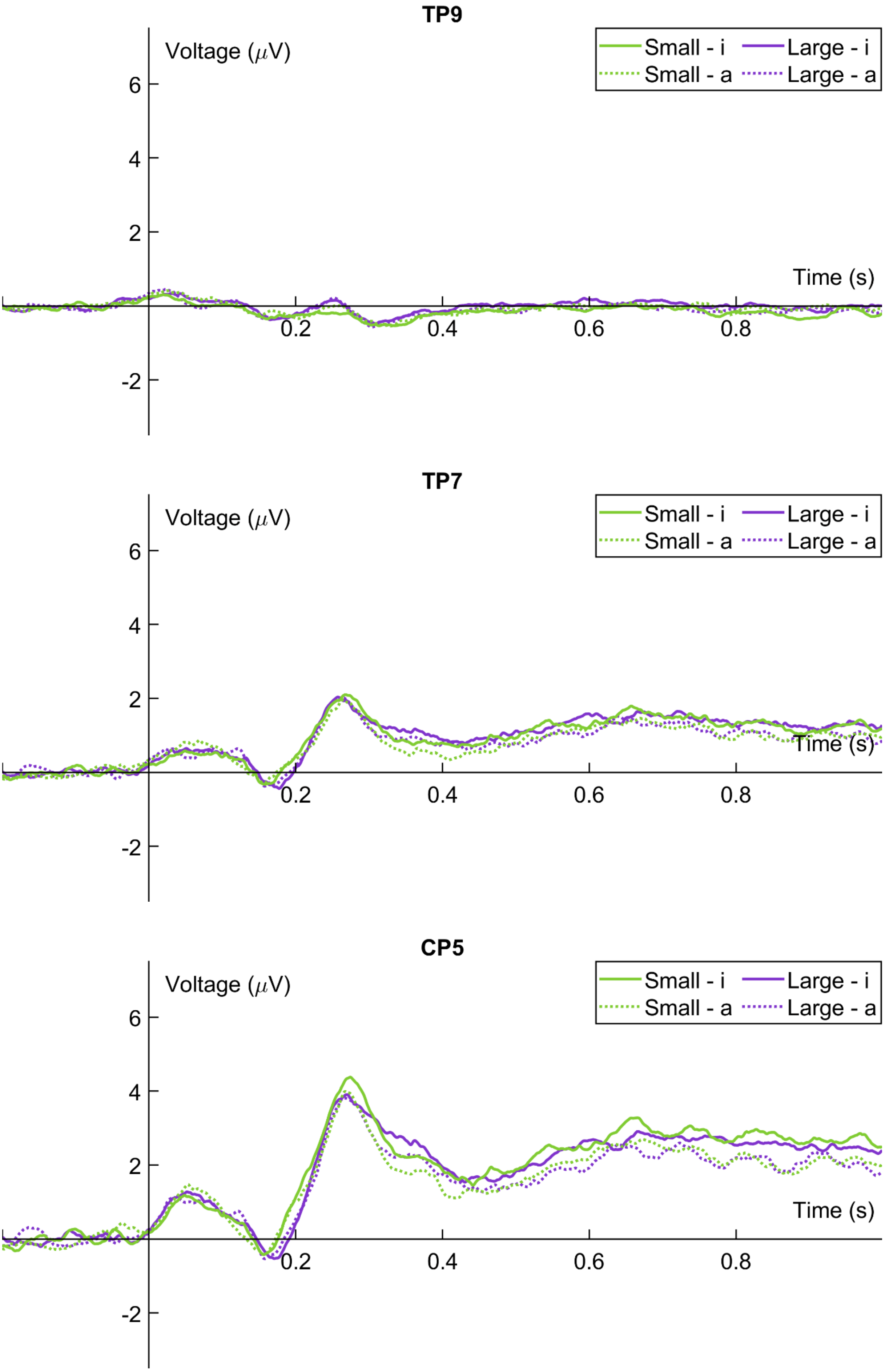

Grand-Average ERPs for all EEG Channels

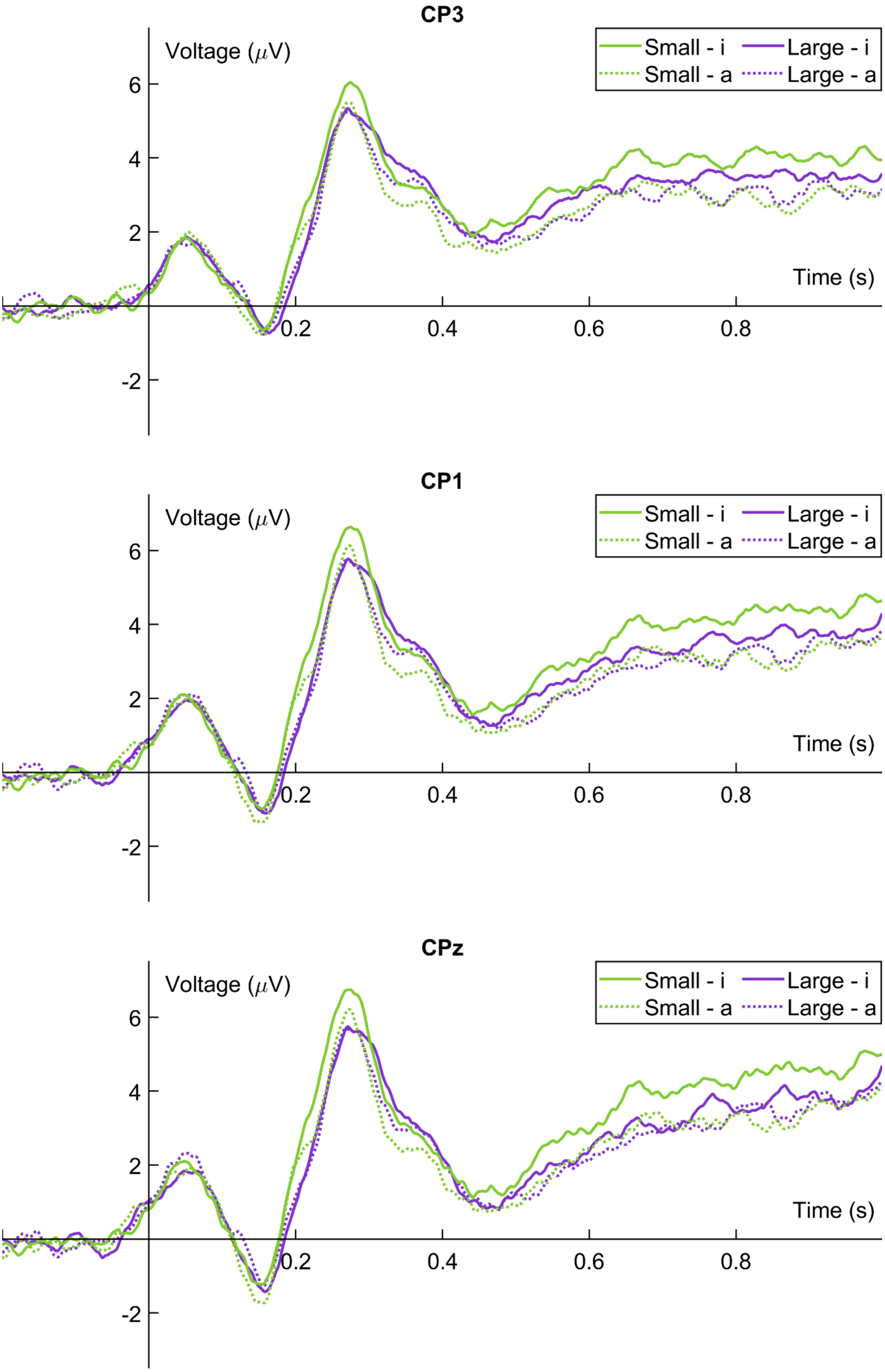

Grand-Average ERPs for all EEG Channels

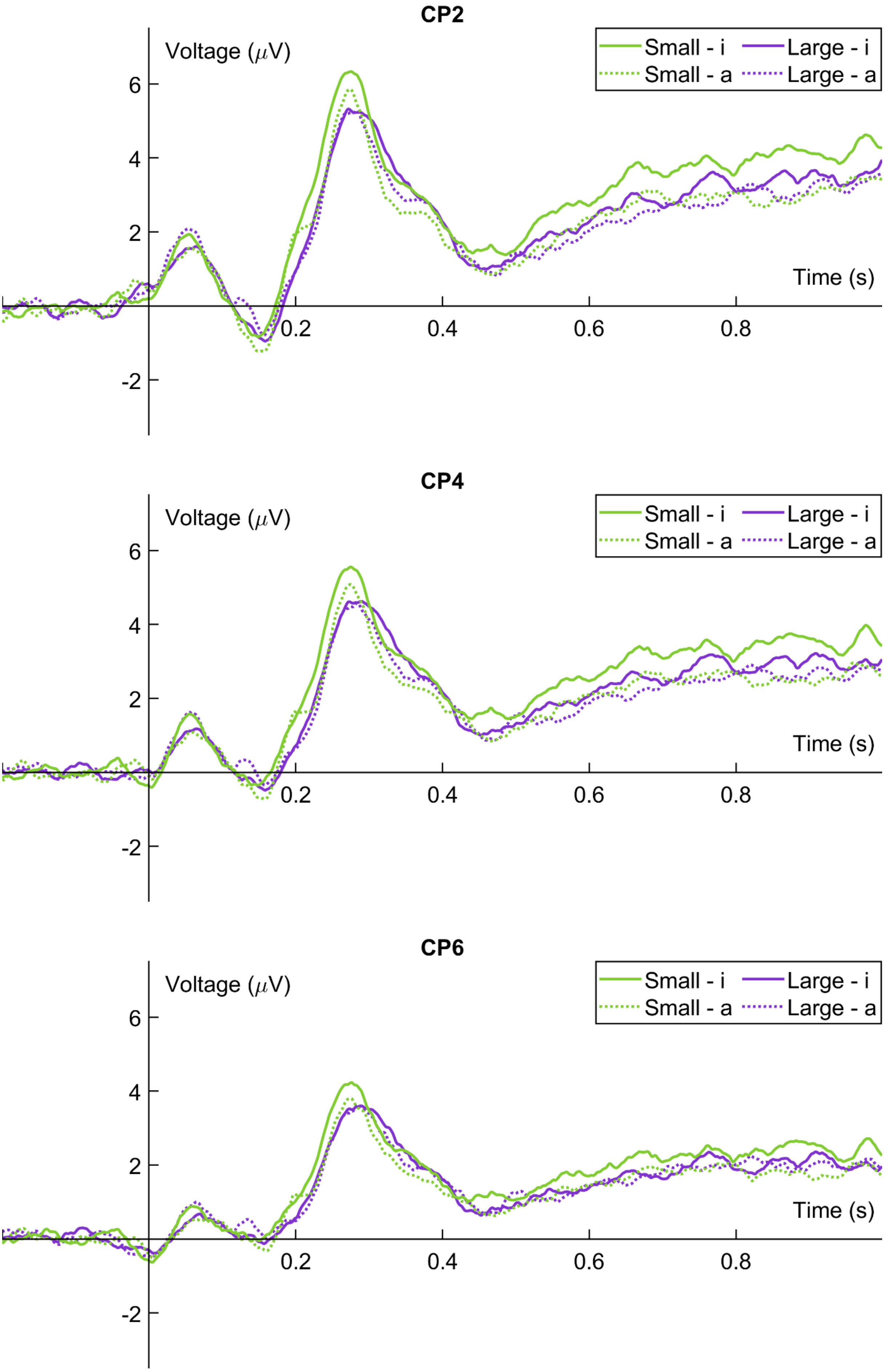

Grand-Average ERPs for all EEG Channels

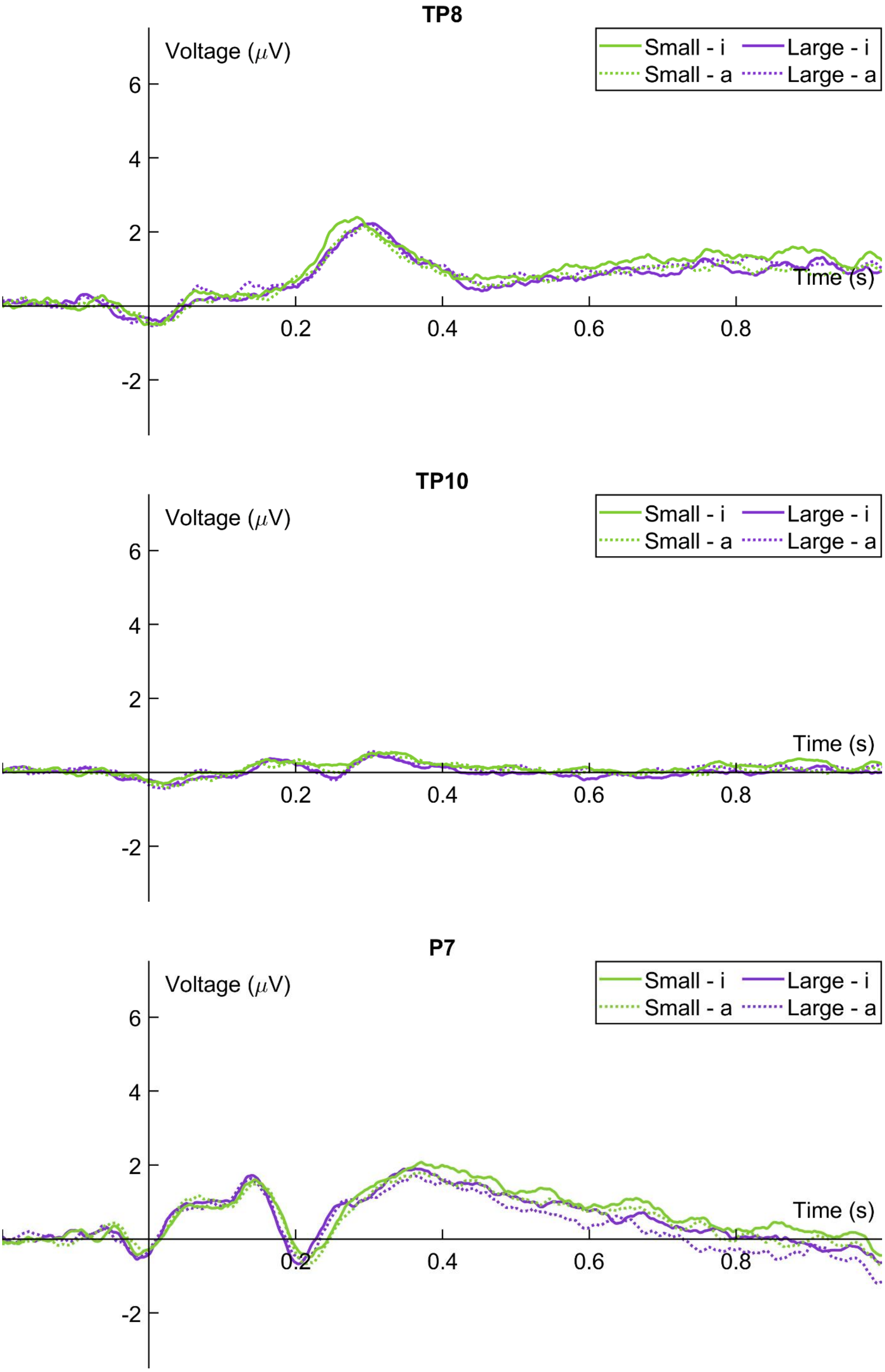

Grand-Average ERPs for all EEG Channels

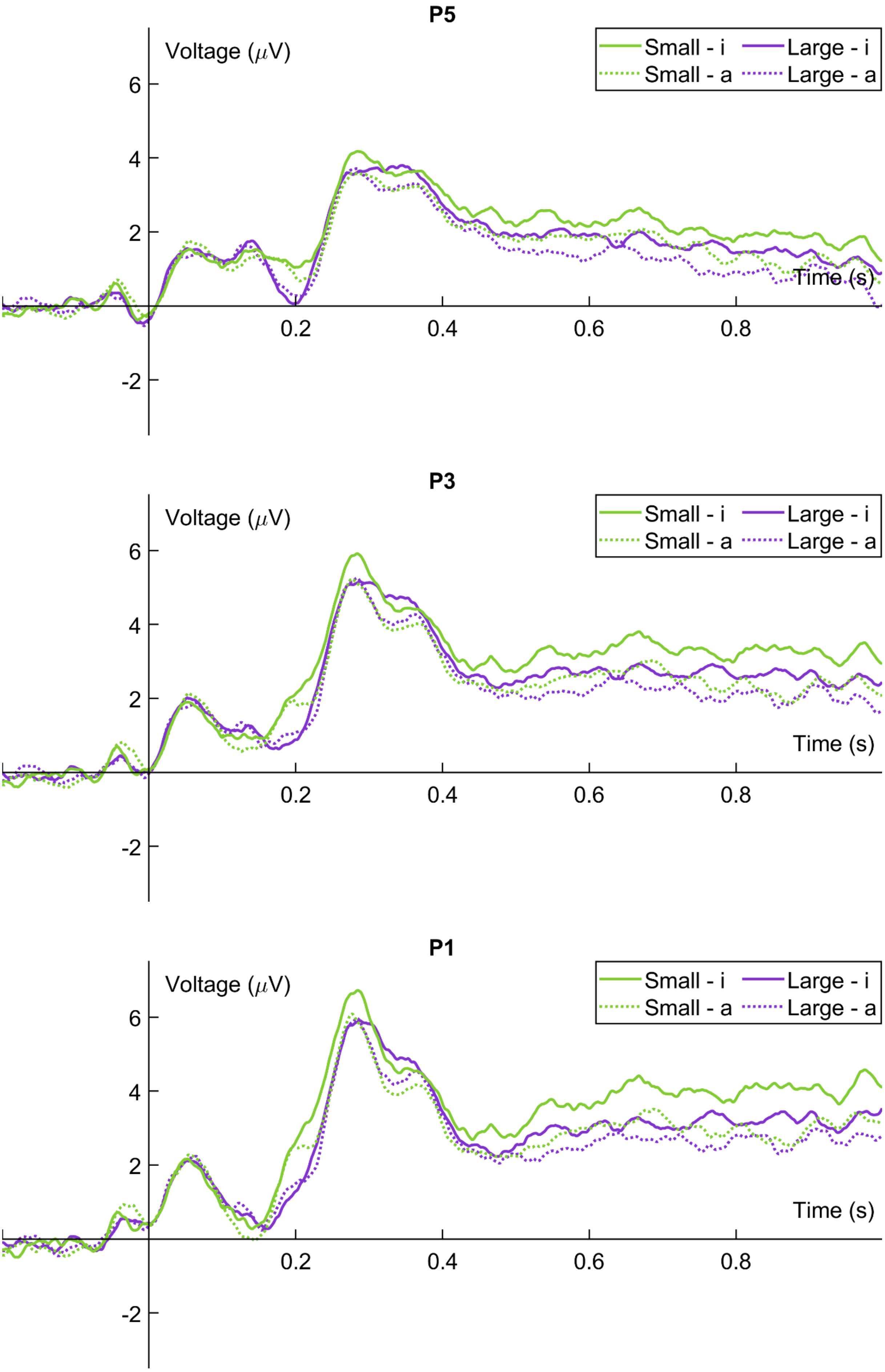

Grand-Average ERPs for all EEG Channels

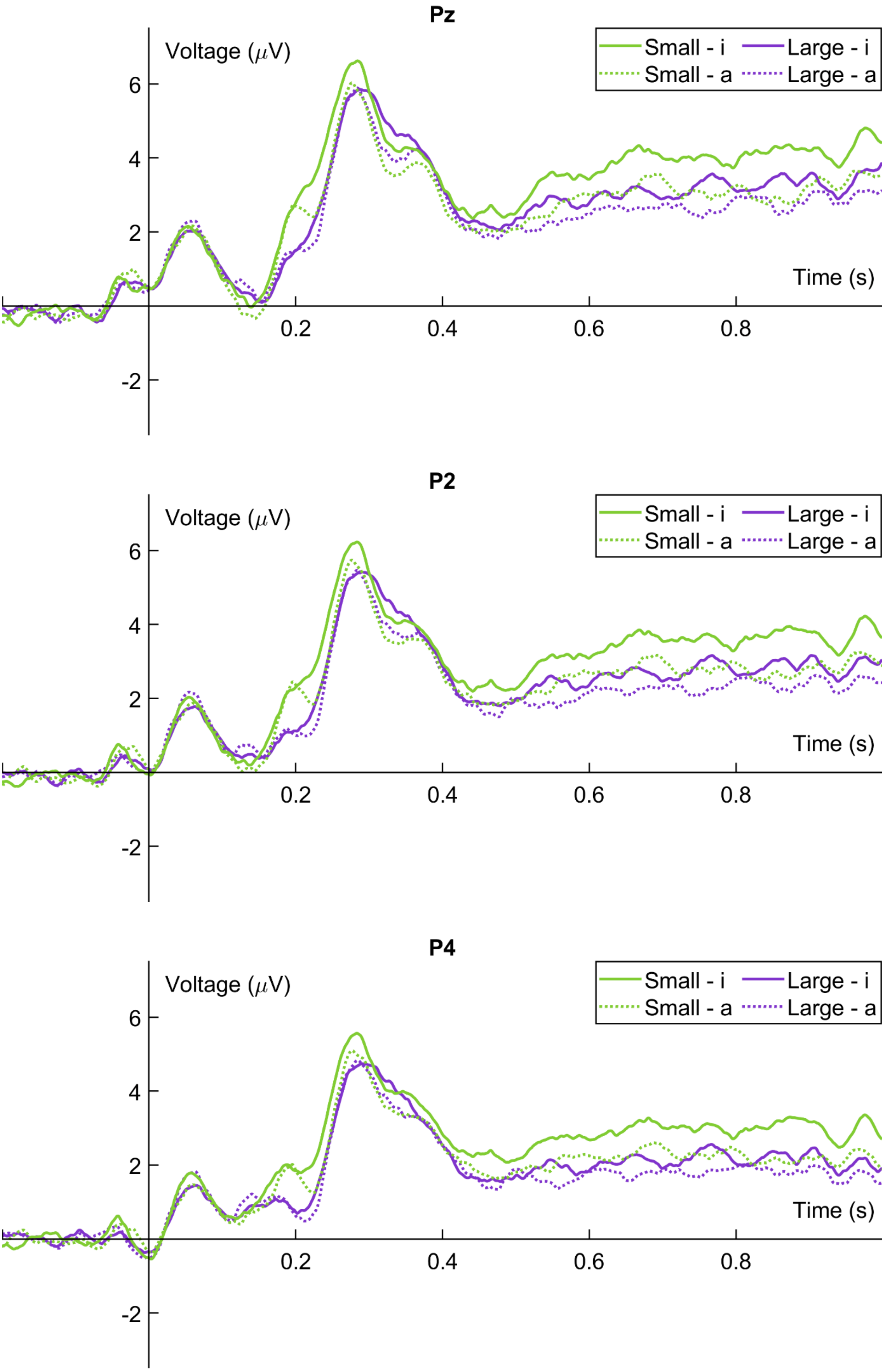

Grand-Average ERPs for all EEG Channels

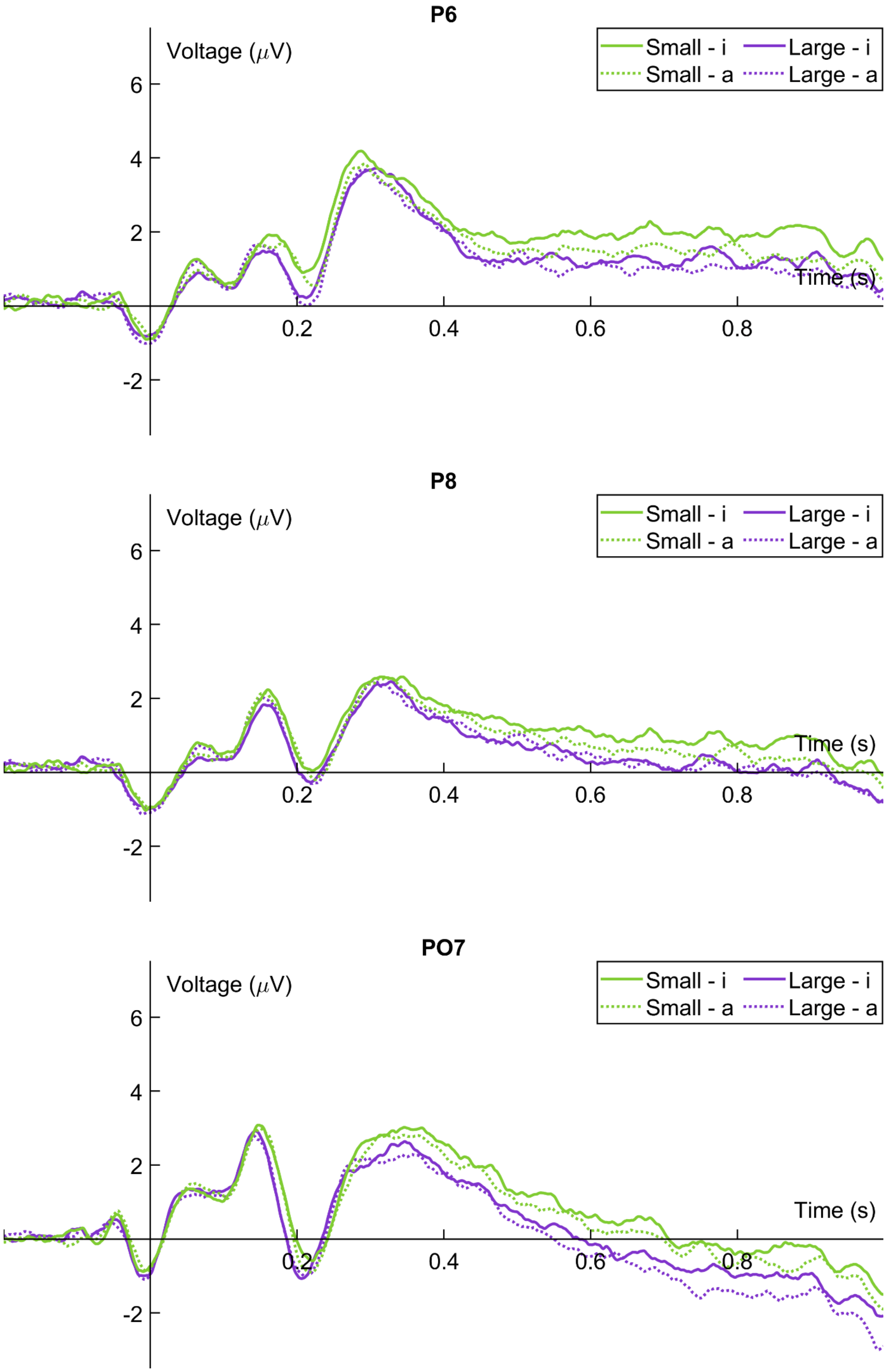

Grand-Average ERPs for all EEG Channels

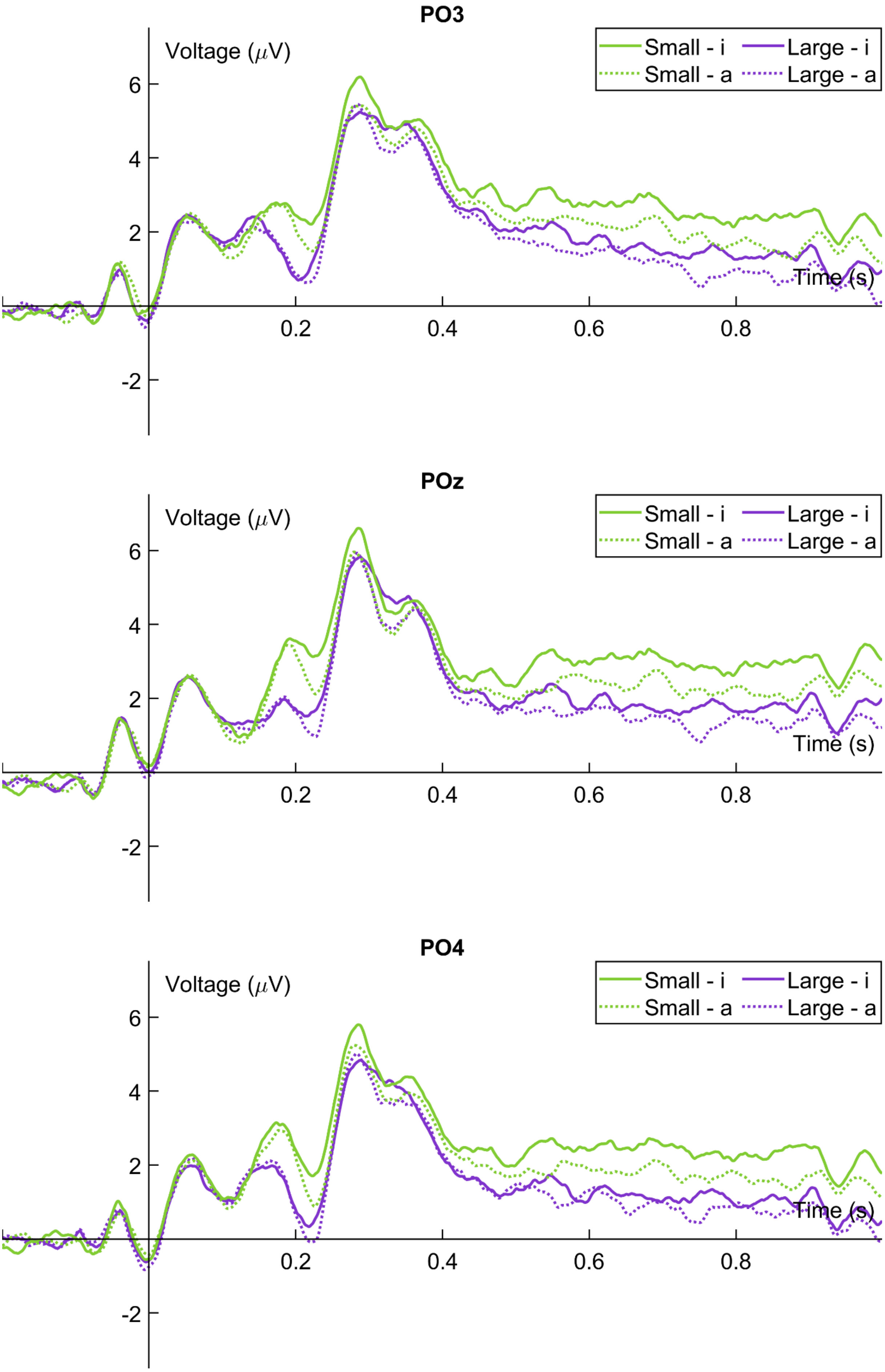

Grand-Average ERPs for all EEG Channels

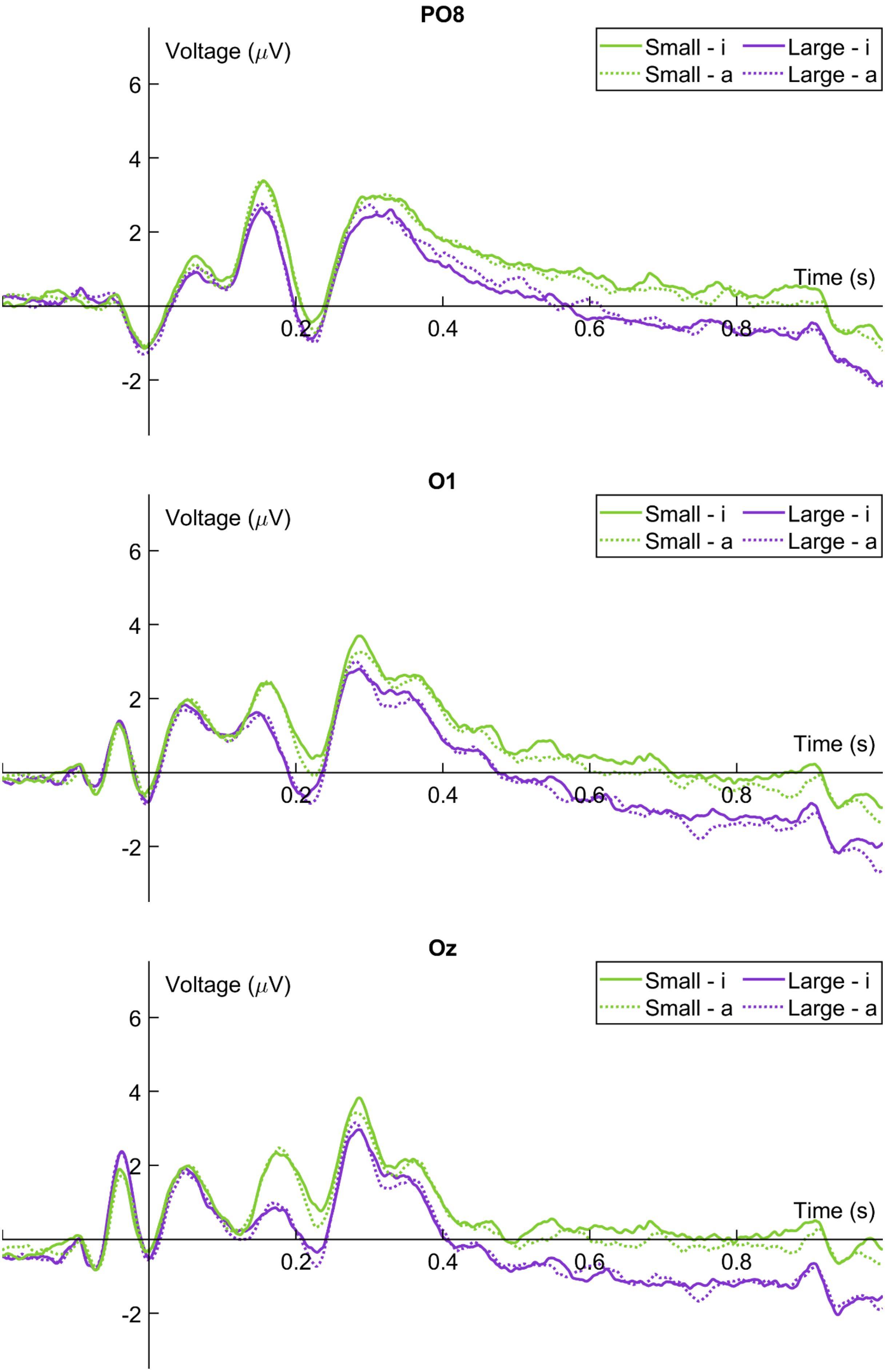

Grand-Average ERPs for all EEG Channels

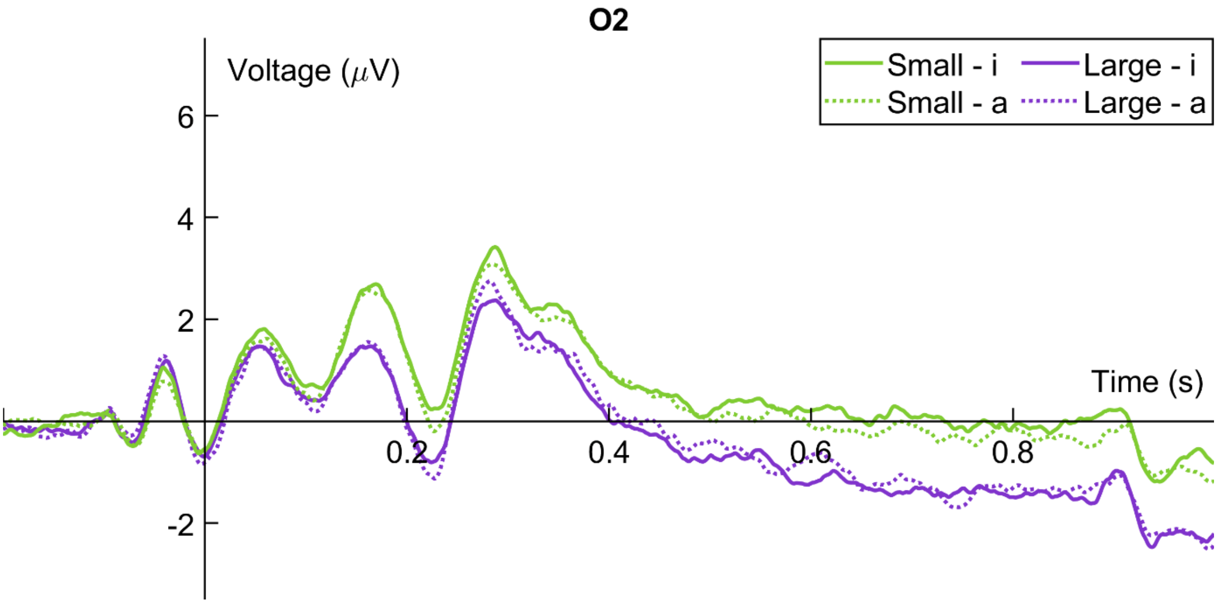

Supplement: Supplementary file 1 — Data S1: psyp70190‐sup‐0001‐Supinfo.zip. [file PSYP-62-e70190-s001.zip › psyp70190-sup-0001-Supinfo.pdf]
